# Emergence of a Plasmid-Encoded Resistance-Nodulation-Division Efflux Pump Conferring Resistance to Multiple Drugs, Including Tigecycline, in Klebsiella pneumoniae

**DOI:** 10.1128/mBio.02930-19

**Published:** 2020-03-03

**Authors:** Luchao Lv, Miao Wan, Chengzhen Wang, Xun Gao, Qiwen Yang, Sally R. Partridge, Yang Wang, Zhiyong Zong, Yohei Doi, Jianzhong Shen, Peiyao Jia, Qianhua Song, Qianhui Zhang, Jun Yang, Xianhui Huang, Minggui Wang, Jian-Hua Liu

**Affiliations:** a College of Veterinary Medicine, National Risk Assessment Laboratory for Antimicrobial Resistance of Microorganisms in Animals, Guangdong Provincial Key Laboratory of Veterinary Pharmaceutics Development and Safety Evaluation, South China Agricultural University, Guangzhou, China; b Guangdong Laboratory for Lingnan Modern Agriculture, Guangzhou, China; c Department of Clinical Laboratory, Peking Union Medical College Hospital, Peking Union Medical College, Chinese Academy of Medical Sciences, Beijing, China; d Centre for Infectious Diseases and Microbiology, The Westmead Institute for Medical Research, The University of Sydney, Westmead Hospital, Sydney, Australia; e Beijing Advanced Innovation Center for Food Nutrition and Human Health, College of Veterinary Medicine, China Agricultural University, Beijing, China; f Center of Infectious Diseases, West China Hospital, Sichuan University, Chengdu, China; g Departments of Microbiology and Infectious Diseases, Fujita Health University School of Medicine, Toyoake, Aichi, Japan; h Division of Infectious Diseases, University of Pittsburgh School of Medicine, Pittsburgh, Pennsylvania, USA; i Institute of Antibiotics, Huashan Hospital, Fudan University, Shanghai, China; Louis Stokes Veterans Affairs Medical Center

**Keywords:** *Enterobacteriaceae*, antimicrobial agents, efflux pumps, mechanisms of resistance, multidrug resistance, plasmid-mediated resistance

## Abstract

Transporters belonging to the chromosomally encoded resistance-nodulation-division (RND) superfamily mediate multidrug resistance in Gram-negative bacteria. However, the cotransfer of large gene clusters encoding RND-type pumps from the chromosome to a plasmid appears infrequent, and no plasmid-mediated RND efflux pump gene cluster has yet been found to confer resistance to tigecycline. Here, we identified a novel RND efflux pump gene cluster, designated *tmexCD1-toprJ1*, on plasmids from five pandrug-resistant Klebsiella pneumoniae isolates of animal origin. TMexCD1-TOprJ1 increased (by 4- to 32-fold) the MICs of tetracyclines (including tigecycline and eravacycline), quinolones, cephalosporins, and aminoglycosides for K. pneumoniae, Escherichia coli, and *Salmonella*. TMexCD1-TOprJ1 is closely related (64.5% to 77.8% amino acid identity) to the MexCD-OprJ efflux pump encoded on the chromosome of Pseudomonas aeruginosa. In an IncFIA plasmid, pHNAH8I, the *tmexCD1-toprJ1* gene cluster lies adjacent to two genes encoding site-specific integrases, which may have been responsible for its acquisition. Expression of TMexCD1-TOprJ1 in E. coli resulted in increased tigecycline efflux and in K. pneumoniae negated the efficacy of tigecycline in an *in vivo* infection model. Expression of TMexCD1-TOprJ1 reduced the growth of E. coli and *Salmonella* but not K. pneumoniae. *tmexCD1-toprJ1*-positive *Enterobacteriaceae* isolates were rare in humans (0.08%) but more common in chicken fecal (14.3%) and retail meat (3.4%) samples. Plasmid-borne *tmexCD1-toprJ1*-like gene clusters were identified in sequences in GenBank from *Enterobacteriaceae* and *Pseudomonas* strains from multiple continents. The possibility of further global dissemination of the *tmexCD1*-*toprJ1* gene cluster and its analogues in *Enterobacteriaceae* via plasmids may be an important consideration for public health planning.

## Abstract (precis)

In an era of increasing concerns about antimicrobial resistance, tigecycline is likely to have a critically important role in the treatment of carbapenem-resistant *Enterobacteriaceae*, the most problematic pathogens in human clinical settings—especially carbapenem-resistant K. pneumoniae. Here, we identified a new plasmid-borne RND-type tigecycline resistance determinant, TMexCD1-TOprJ1, which is widespread among K. pneumoniae isolates from food animals. *tmexCD1-toprJ1* appears to have originated from the chromosome of a *Pseudomonas* species and may have been transferred onto plasmids by adjacent site-specific integrases. Although *tmexCD1-toprJ1* still appears to be rare in human clinical isolates, considering the transferability of the *tmexCD1-toprJ1* gene cluster and the broad substrate spectrum of TMexCD1-TOprJ1, further dissemination of this mobile tigecycline resistance determinant is possible. Therefore, from a “One Health” perspective, measures are urgently needed to monitor and control its further spread. The current low prevalence in human clinical isolates provides a precious time window to design and implement measures to tackle this.

## INTRODUCTION

The increasing occurrence of multidrug-resistant (MDR), Gram-negative bacteria, and especially of extensively drug-resistant (XDR) and pandrug-resistant (PDR) strains, presents a serious threat to global public health (1, 2). Lateral gene transfer mediated by mobile genetic elements, such as plasmids, is a key factor contributing to the emergence and global dissemination of MDR bacteria (3). Chromosomally encoded tripartite efflux pumps of the resistance-nodulation-division (RND) superfamily mediate multidrug resistance in Gram-negative bacteria (4, 5). RND family exporters usually require three gene products to function effectively. Consequently, cotransfer of entire gene clusters encoding RND-type tripartite drug efflux pumps from the chromosome to a plasmid appears rare to date (4, 6).

Management of infections caused by MDR Gram-negative bacteria, particularly carbapenem-resistant *Enterobacteriaceae* (CRE), is a major challenge due to limited treatment options (7). Recently, the WHO listed CRE as critical-priority pathogens for which investment in the development of new drugs is urgently needed (1). Polymyxins (colistin or polymyxin B) and tigecycline have been regarded as mainstream treatment options for infections caused by CRE (7). However, the emergence and global dissemination of plasmid-borne colistin resistance genes threatens to compromise the efficacy of polymyxins (8), leaving tigecycline as one of the few drugs of choice for treating serious CRE infections. As a semisynthetic parenteral glycylcycline with a broad spectrum of antimicrobial activity, tigecycline can circumvent tetracycline resistance mediated by active efflux pumps or ribosomal protection in *Enterobacteriaceae* (9). However, tigecycline-resistant *Enterobacteriaceae* are increasingly reported following the widespread clinical use of tigecycline, especially against carbapenem-resistant Klebsiella pneumoniae (CRKP), the most common type of CRE (10). Importantly, the prevalence of CRKP has increased steadily worldwide over the last decade (11, 12). Not surprisingly, infections caused by CRKP are very difficult to treat, resulting in high mortality rates (>40%) (2, 13).

The mechanisms underlying tigecycline resistance in *Enterobacteriaceae* are complicated and have not been fully elucidated. In K. pneumoniae, overexpression of the intrinsic chromosomally encoded RND-type efflux pumps AcrAB and OqxAB, which results from mutations in transcriptional regulator genes (*ramR* and *acrR*), has been shown to play an important role in tigecycline resistance (14–16). The *oqxAB* genes are also found on plasmids in other species, and there is a report of *oqxAB* contributing to tigecycline heteroresistance in *Salmonella* (17). Mutations in *rpsJ*, encoding the ribosomal S10 protein targeted by tetracyclines, are also associated with decreased tigecycline susceptibility (18). In addition to chromosomal mechanisms, mutations in the plasmid-borne tetracycline efflux pump gene *tet*(A) reportedly contribute to tigecycline resistance (19). Very recently, plasmid-borne genes *tet*(X3), *tet*(X4), and *tet*(X5), encoding the tigecycline modification enzymes, were reported in Acinetobacter baumannii and *Enterobacteriaceae* from China (20–22).

Although overexpression of chromosomal RND-type efflux pumps like AcrAB-TolC, MexXY-OprM, and OqxAB in Gram-negative bacteria results in reduced susceptibility to tigecycline (4, 23), and genes encoding RND-type efflux pumps have been identified on plasmids (6, 24–27), little is known about the involvement of plasmid-encoded RND-type efflux pumps in tigecycline resistance. Here, we identified a plasmid-borne gene cluster, *tmexCD1*-*toprJ1*, encoding an RND family multidrug efflux pump that confers resistance to tetracyclines, including tigecycline and eravacycline, as well as reduced susceptibility to multiple clinically important antimicrobials (quinolones, cephalosporins, and aminoglycosides). Our findings suggest that *tmexCD1*-*toprJ1* has been transferred from the chromosome of a *Pseudomonas* species to a plasmid, apparently by adjacent site-specific integrases.

## RESULTS

### Characterization of tigecycline-resistant K. pneumoniae isolates.

In a project investigating CRE carriage by food-producing animals in China, we identified five pandrug-resistant K. pneumoniae isolates (AH6I, AH8I, AH25I, AH28I, and AH33I) from fecal samples of five healthy 12-day-old chickens from a chicken farm in Anhui province in 2017. The five isolates were resistant or nonsusceptible to all antibiotics tested, including tigecycline (MIC, 16 mg/liter) (Table 1; Table S1 in the supplemental material). They all were classified as sequence type 1 (ST1) (Table S2) and gave highly similar pulsed-field gel electrophoresis (PFGE) patterns (Fig. S1). Whole-genome sequencing (WGS) of one isolate, AH8I, revealed 30 known resistance genes consistent with observed resistance or reduced susceptibility to β-lactams (*bla*_NDM-1_), colistin (*mcr-1.1* and *mcr-8.1*), aminoglycosides (*armA*), quinolones, tetracyclines [*tet*(A)], and florfenicol (*floR*) (Table S3). Using PCR assays and Sanger sequencing, we confirmed that the other four isolates also contained *bla*_NDM-1_, *mcr-1.1*, *mcr-8.1*, and *armA* genes. Known tigecycline resistance mechanisms [mutations in *ramR*, *acrR*, *rpsJ*, or *tet*(A)] were not identified in AH8I, but addition of the efflux pump inhibitor 1-(1-naphthylmethyl)-piperazine (NMP) reduced the tigecycline MIC against all five isolates by 64- to 128-fold (Table 1).

**TABLE 1:** MICs against parental strains, transformants, and transconjugants

Strain(s)	MIC (mg/liter) of^*a*^:													
	TIG (+NMP)	ERV	DOX	TET	MIN	OXY	CTC	CTX	CAZ	CQM	FEP	CIP	STR	GEN
Pandrug-resistant *K. pneumoniae* strains														
AH8I, AH6I, AH25I, AH28I, and AH33I^*b*^	16 (<0.0156)	32	64	>128	64	>128	>128	>128	>128	>128	>32	>64	>128	>128
*E. coli* strains														
J53	0.5	0.5	4	1	4	8	4	0.03	0.25	0.03	0.03	0.008	1	0.125
J53/pHNAH8I-1	4	4	16	8	16	16	16	0.06	0.25	0.5	0.25	0.03	8	0.25
DH5α	0.25	0.5	2	1	2	2	4	0.03	0.125	0.03	0.03	0.002	1	0.125
DH5α/pHNAH8I-1	8	16	32	32	32	64	32	0.5	1	2	0.25	0.03	128	4
DH5α/pHSG575	0.25	0.5	2	1	2	4	4	0.03	0.125	0.03	0.03	0.002	1	0.125
DH5α/pHSG575-tmexCD1	1	2	4	4	4	8	16	0.125	0.25	0.25	0.25	0.0156	4	0.25
DH5α/pHSG575-tnfxB1-tmexCD1	1	2	4	4	4	8	16	0.125	0.25	0.25	0.25	0.0156	4	0.25
DH5α/pHSG575-tmexCD1-toprJ1	4	8	32	16	16	16	32	0.25	1	1	0.5	0.03	16	1
DH5α/pHSG575-tnfxB1-tmexCD1- toprJ1	4	8	32	16	16	16	32	0.25	1	1	0.5	0.03	16	1
*K. pneumoniae* strains														
YX94	0.5	1	2	2	4	4	8	0.03	0.06	0.06	0.06	0.008	8	0.25
YX94/pHNAH8I-1	8	16	16	8	16	16	16	0.125	0.5	2	1	0.25	>128	1
YX94/pHSG575	0.5	1	2	2	4	8	4	0.03	0.06	0.06	0.06	0.008	8	0.25
YX94/pHSG575-tnfxB1-tmexCD1- toprJ1	16	16	32	32	32	32	16	0.125	0.5	2	0.5	0.25	>128	4
AH58I	0.5	1	4	8	4	8	4	>128	>128	>128	>32	32	16	4
AH58I/pHNAH8I-1	8	16	32	32	16	32	16	>128	>128	>128	>32	64	>128	16
*S.* Typhimurium strains														
HN227/pHSG575	0.5	1	4	2	4	8	4	0.125	0.5	0.125	0.06	0.008	8	0.25
HN227/pHSG575-tnfxB1- tmexCD1-toprJ1	16	16	32	32	32	128	32	0.5	2	8	4	0.25	128	4

a TIG, tigecycline; ERV, eravacycline; DOX, doxycycline; TET, tetracycline; MIN, minocycline; OXY, oxytetracycline; CTC, chlortetracycline; CTX, cefotaxime; CAZ, ceftazidime; CQM, cefquinome; FEP, cefepime; CIP, ciprofloxacin; STR, streptomycin; GEN, gentamicin. MICs of tigecycline were determined by microdilution and those of other antibiotics by agar dilution. NMP was used with all the tested antibiotics, but the results are not shown.

b The imipenem MICs against the five K. pneumoniae isolates were all 8 mg/liter, and MICs of fosfomycin were all 32 mg/liter.

10.1128/mBio.02930-19.1 FIG S1 PFGE patterns of five tigecycline-resistant Klebsiella pneumoniae isolates from chickens. Download FIG S1, TIF file, 0.5 MB. Copyright © 2020 Lv et al. 2020 Lv et al. This content is distributed under the terms of the Creative Commons Attribution 4.0 International license.

10.1128/mBio.02930-19.5 TABLE S1 MICs against parental strain, transformants, and transconjugant (mg/liter). Download Table S1, DOCX file, 0.02 MB. Copyright © 2020 Lv et al. 2020 Lv et al. This content is distributed under the terms of the Creative Commons Attribution 4.0 International license.

10.1128/mBio.02930-19.6 TABLE S2 Isolates used in this study. Download Table S2, DOCX file, 0.02 MB. Copyright © 2020 Lv et al. 2020 Lv et al. This content is distributed under the terms of the Creative Commons Attribution 4.0 International license.

10.1128/mBio.02930-19.7 TABLE S3 Resistance genes and plasmids carried by Klebsiella pneumoniae AH8I. Download Table S3, DOCX file, 0.02 MB. Copyright © 2020 Lv et al. 2020 Lv et al. This content is distributed under the terms of the Creative Commons Attribution 4.0 International license.

### Identification of a novel plasmid-borne *nfxB-mexCD-oprJ*-like gene cluster.

Long-read sequencing of isolate AH8I using the PacBio RSII system allowed complete assembly of the chromosome and five plasmids (Table S3). One plasmid, designated pHNAH8I-1, carries *mcr-8.1*, *strAB*, and a 6.5-kb region containing a cluster of genes encoding homologs of a tripartite efflux pump with 64.5% to 77.8% amino acid identity to MexCD-OprJ of Pseudomonas aeruginosa strain PAO1. This gene cluster is adjacent to an open reading frame annotated as *nfxB*, which encodes a transcriptional regulator (28). Because this gene cluster is plasmid borne and transferrable, we named it *tnfxB1-tmexCD1-toprJ1* (“t” for transferrable). Alignment of the encoded proteins (TMexC1, TMexD1, and TOprJ1) with those of three well-characterized RND efflux pumps conferring multidrug resistance (MexCD-OprJ and MexAB-OprM from P. aeruginosa PAO1 and AcrAB-TolC from Escherichia coli strain K-12) (28, 29) predicted that all proteins would have secondary structures nearly identical to those of their respective homologues (Fig. S2 to Fig. S4). In addition, TMexD1 contains all 12 transmembrane segments common to most RND proteins (Fig. S3), further supporting the hypothesis that TMexCD1-TOprJ1 is an RND family efflux pump.

10.1128/mBio.02930-19.2 FIG S2 Amino acid sequence alignments and predicted secondary structures of TMexC1 from Klebsiella pneumoniae AH8I and homologs. Alignments were generated with ClustalX 2 software then visualized using the ESPript server (http://espript.ibcp.fr/ESPript/cgi-bin/ESPript.cgi). Identical and similar residues are boxed in red and blue, respectively. Secondary structure is shown on the lines above the sequence alignment with α-helices marked by coils, β-strands by arrows, and strict β-turns by TT or TTT. The PDB IDs are as follows: NP_249116.1 (MexA); NP_253289.1 (MexC); NP_414996.1 (AcrA). The last letter (P or C) of each protein name indicates the location of the coding sequence (P, encoded on a plasmid; C, encoded on the chromosome). Download FIG S2, TIF file, 2.0 MB. Copyright © 2020 Lv et al. 2020 Lv et al. This content is distributed under the terms of the Creative Commons Attribution 4.0 International license.

To determine whether *tnfxB1-tmexCD1-toprJ1* could confer resistance to tigecycline, we constructed four recombinant plasmids: pHSG575-tnfxB1-tmexCD1-toprJ1, pHSG575-tmexCD1-toprJ1 (missing *tnfxB1*), pHSG575-tnfxB1-tmexCD1 (missing *toprJ1*), and pHSG575-tmexCD1 (missing both *tnfxB1* and *toprJ1*). The MIC of tigecycline was 16-fold higher for E. coli strain DH5α transformed with pHSG575-tnfxB1-tmexCD1-toprJ1 or pHSG575-tmexCD1-toprJ1 than for E. coli DH5α carrying pHSG575 alone (Table 1). The presence of either of these plasmids also resulted in 8- to 32-fold-higher MICs for six other tetracyclines, including eravacycline. Reduced susceptibility was also observed for five β-lactams, including ceftazidime, and cefepime (8- to 32-fold increases in MICs), quinolones (nalidixic acid and ciprofloxacin; 8- to 16-fold increases in MICs), aminoglycosides (streptomycin and gentamicin; 8- to 16-fold increases in MICs), and phenicols (florfenicol; 16-fold increase in MIC). Transformation with pHSG575-tnfxB1-tmexCD1 and pHSG575-tmexCD1 also increased the MICs of these antimicrobials by 2- to 16-fold, although the increases were 2- to 8-fold lower than those observed for strains carrying pHSG575-tnfxB1-tmexCD1-toprJ1 and pHSG575-tmexCD1-toprJ1. When transferred into naturally occurring strains susceptible to all antimicrobials tested (K. pneumoniae strain YX94 and Salmonella enterica serovar Typhimurium strain HN227) (Table 1; Table S1), pHSG575-tnfxB1-tmexCD1-toprJ1 increased the tigecycline MICs by 16- to 32-fold and reduced the susceptibility of strains YX94 and HN227 to the other antimicrobials (4- to 32-fold increases in MICs) (Table 1).

### Tigecycline accumulation in E. coli expressing TMexCD1-TOprJ1.

**FIG 1:**
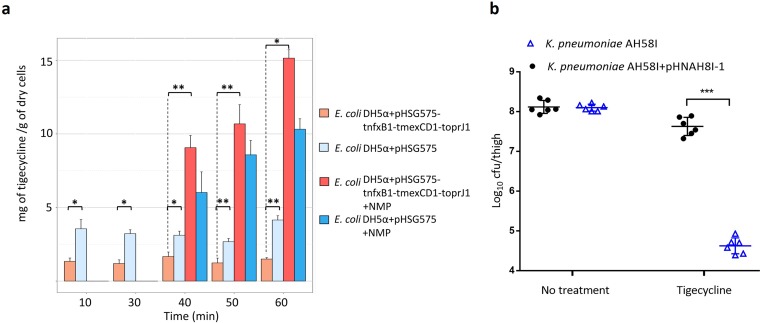
The activity of TMexCD1-TOprJ1 on tigecycline *in vitro* and *in vivo*. (a) Tigecycline accumulation by E. coli DH5α carrying pHSG575 or pHSG575-tnfxB1-tmexCD1-toprJ1. Each bar and error bar show the mean value and standard deviation of three replicates. Student’s *t* tests were performed to analyze data. *, *P* < 0.05; **, *P* < 0.01. (b) *In vivo* effects of plasmid pHNAH8I-1 carrying *tmexCD1-toprJ1* on the efficacy of tigecycline treatment in a neutropenic-mouse thigh infection model. Error bars represent the standard deviations of the means (*n* = 6). ***, *P* < 0.001.

To confirm the function of TMexCD1-TOprJ1 as an efflux pump, the intracellular accumulation of tigecycline in E. coli DH5α carrying pHSG575-tnfxB1-tmexCD1-toprJ1 or control plasmid pHSG575 was determined. At most time points, the average drug concentration in the control strain was approximately 2-fold higher than that in the strain containing pHSG575-tnfxB1-tmexCD1-toprJ1 (*P* < 0.05) (Fig. 1a). After the addition of the efflux pump inhibitor NMP, rapid increases in intracellular tigecycline accumulation were observed in both the control (2-fold increase) and the study strain expressing TMexCD1-TOprJ1 (7-fold increase). Constitutively expressed RND efflux pump(s) (e.g., AcrAB-TolC) in E. coli may be responsible for the elevated drug concentration in the E. coli DH5α/pHSG575 control strain following treatment with NMP (30), while the increased accumulation in the TMexCD1-TOprJ1 overexpression strain may result from repression of other pumps that would usually help to efflux tigecycline. Taken together, these data confirm that TMexCD1-TOprJ1 functions as a drug efflux pump.

### Impact of TMexCD1-TOprJ1 on the effects of tigecycline treatment *in vivo*.

To evaluate whether TMexCD1-TOprJ1 affects the therapeutic effects of tigecycline *in vivo*, tigecycline-sensitive K. pneumoniae strain AH58I or the same strain with the *tmexCD1-toprJ1*-bearing plasmid pHNAH8I-1 was injected into mouse thigh muscles. Following 36 h of treatment with tigecycline, a 3-log decrease in the concentration of AH58I cells was observed, compared with a 0.5-log decrease in AH58I/pHNAH8I-1 cells (Fig. 1b). This finding indicates that TMexCD1-TOprJ1 does indeed confer resistance to tigecycline and may lead to tigecycline treatment failure in humans.

### Impact of TMexCD1-TOprJ1 on the growth of bacteria.

**FIG 2:**
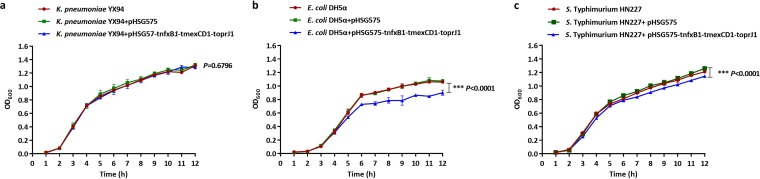
Growth curves of K. pneumoniae YX94, E. coli DH5α, *S.* Typhimurium HN227, and their transformants. Values represent the means ± standard deviations obtained from three independent repeated experiments. (a) Optical densities of K. pneumoniae YX94 and its transformants at 600 nm (OD_600_) measured at hourly intervals. (b) Optical densities of E. coli DH5α and its transformants. (c) Optical densities of *S.* Typhimurium HN227 and its transformants. A repeated measures two-way analysis of variance (ANOVA) with Tukey’s multiple comparison was used to evaluate statistical significance.

To assess the impact of TMexCD1-TOprJ1 expression on the growth of *Enterobacteriaceae* strains, we compared the growth of E. coli DH5α, K. pneumoniae YX94, and *S.* Typhimurium HN227 with the growth of their corresponding transformants carrying pHSG575 or pHSG575-tnfxB1-tmexCD1-toprJ1.The growth curves of K. pneumoniae YX94, K. pneumoniae YX94/pHSG575, and K. pneumoniae YX94/pHSG575-tnfxB1-tmexCD1-toprJ1 were almost identical, with all strains showing similar lag- and logarithmic-phase profiles (Fig. 2a). However, the growth of E. coli DH5α/pHSG575-tnfxB1-tmexCD1-toprJ1 and *S.* Typhimurium HN227/pHSG575-tnfxB1-tmexCD1-toprJ1 was clearly (*P* < 0.001) impaired, as evidenced by decreases in optical density compared with the optical densities of E. coli DH5α/pHSG575 and *S.* Typhimurium HN227/pHSG575, respectively (Fig. 2b and c). These results indicate that the expression of TMexCD1-TOprJ1 had no effect on the growth of K. pneumoniae but reduced the growth of E. coli and *S.* Typhimurium.

### Possible origin of *tnfxB1-tmexCD1-toprJ1*.

**FIG 3:**
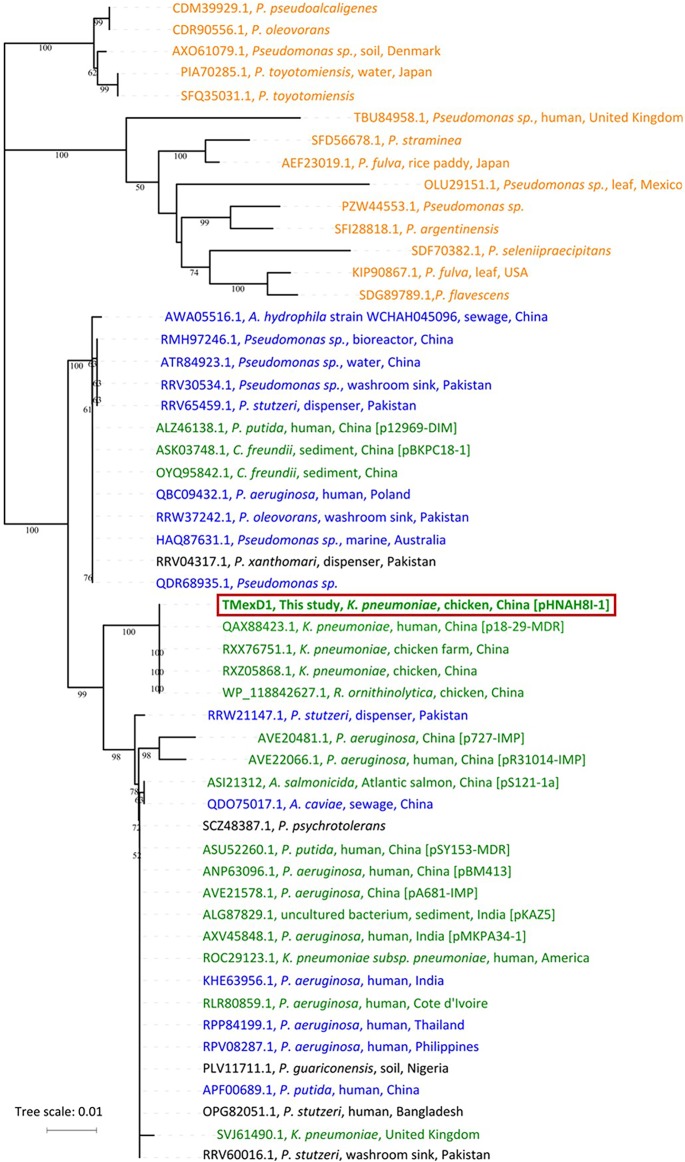
Phylogenetic tree of TMexD1 and TMexD1-like proteins. Neighbor-joining tree based on the amino acid sequences of RND family proteins related to TMexD1 (53 sequences with >90% amino acid identity) generated using MEGA X with 1,000 bootstrap replicates. All amino acid sequences were obtained from the NCBI databases. The tree was visualized using iTOL. The locations of genes encoding the proteins are represented in different colors as follows: orange (chromosome); green (plasmid); blue (chromosome); and black (could not be identified as chromosome or plasmid); the latter two with adjacent mobile genetic elements and showing <90% amino acid sequence identity to the chromosomally encoded MexD protein from the same *Pseudomonas* species, suggesting that these are acquired rather than intrinsic genes.

To elucidate the origin and evolutionary relationships of the *tnfxB1*-*tmexCD1*-*toprJ1* RND efflux complex, we compared TMexD1, the main functional transporter in this tripartite drug efflux pump, with related proteins in GenBank. The resulting neighbor-joining tree places TMexD1 in a phylogenetic group with high amino acid identity (>90%) (Fig. 3). This group includes proteins encoded by 14 chromosomally located genes from *Pseudomonas* species, together with 18 plasmid-borne genes, suggesting that *tmexCD1-toprJ1* may have originated from a certain *Pseudomonas* species. The group also contained 13 proteins from *Pseudomonas* spp. that, although encoded by genes located on the chromosome, were surrounded by mobile genetic elements and showed <90% amino acid sequence identity to the chromosomally encoded MexD protein from the same *Pseudomonas* species, suggesting acquisition from other *Pseudomonas* species (Fig. 3). Of note, 18 TMexD1-like proteins are encoded by plasmids in *Enterobacteriaceae* and *Pseudomonas* strains from multiple countries, including China, India, Cote d’Ivoire, the United States, and the United Kingdom.

### Characterization of pHNAH8I-1 carrying *tmexCD1*-*toprJ1*.

**FIG 4:**
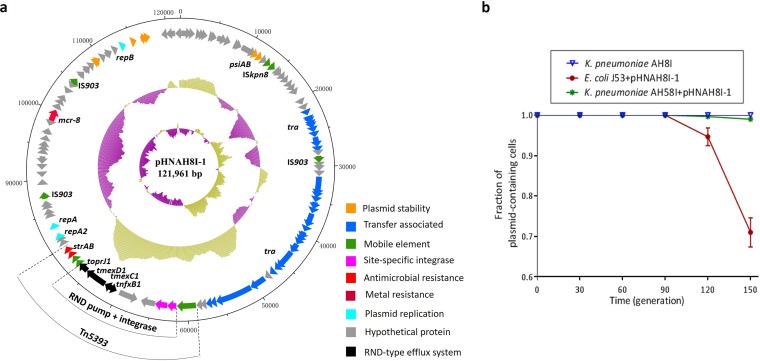
Plasmid structures and stability of pHNAH8I-1. (a) Plasmid pHNAH8I-1. Circles from outside to inside represent sequence positions in base pairs, the locations of predicted forward coding sequences (CDS), %GC plot, and GC skew [(GC)/(G+C)], respectively. The extents and positions of Tn*5393* and the *nfxB1*-*tmexCD1-toprJ1* insertion are shown. Functions encoded by different genes are shown in different colors as indicated in the key. (b) Stability of pHNAH8I-1 in K. pneumoniae AH8I and transconjugants of E. coli J53 and K. pneumoniae AH58I. Error bars represent standard deviations (*n* = 3).

The sequence of pHNAH8I-1 carrying *tnfxB1*-*tmexCD1*-*toprJ1* was also analyzed. pHNAH8I-1 (121,961 bp) contains an FIA replicon and is most closely related (81 to 98% identity in shared regions) to pKP91 from K. pneumoniae isolate KP91 from pig feces in China (GenBank accession number MG736312), with both plasmids containing *mcr-8.1* (Fig. 4a). pHNAH8I-1 was successfully transferred from K. pneumoniae AH8I to K. pneumoniae AH58I (susceptible to tigecycline only) and azide-resistant E. coli strain J53 by conjugation (frequency of ∼10^−6^ to 10^−7^/recipient). In the absence of tigecycline, pHNAH8I-1 was stable for ≥15 days (i.e., ∼150 generations) in K. pneumoniae AH8I and AH58I but was gradually lost from an E. coli J53 transconjugant after 9 days of passage, with 71% retention after 15 days (Fig. 4b). Thus, in the absence of selection, pHNAH8I-1 may be stable in K. pneumoniae but is less stable in E. coli.

### Genetic context and mobilization of *tnfxB1-tmexCD1-toprJ1*.

**FIG 5:**
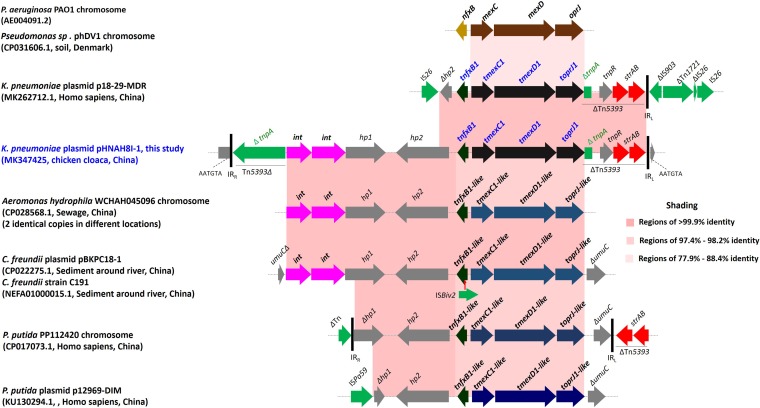
Comparison of the genetic context of *tmexCD1-toprJ1* with those of closely related sequences. The extents and directions of genes are shown by arrows labeled with gene names, with *tmexCD1*-*toprJ1* genes shown as black or blue arrows and *int* indicating genes predicted to encode site-specific integrases. IS*Bvi1* is shown as a green arrow, with the red vertical arrow indicating the insertion point. Tall bars represent the inverted repeats (IRs) of Tn*5393* and other transposons. Horizontal dotted lines represent the plasmid backbone or chromosome. In pHNAH8I-1 and some other sequences, possible 5-bp direct repeats (DRs) were identified flanking the insertion, but comparison with uninterrupted versions of the flanking sequences indicated that DRs are not consistently found adjacent to the same point and the creation of DRs is not generally characteristic of site-specific integration, so these are not shown. The context in p18-29-MDR suggests IS*26*-mediated movement. Additional contigs matching part of the p18-29-MDR context (accession numbers QFMF01000058, QFRE01000039, and QFMA01000080, from K. pneumoniae) or the pHNAH8I context (accession numbers QWTW01000047 and QWIX01000043, from R. ornithinolytica) were also identified, all from chicken cloacae in China. P. putida, Pseudomonas putida.

In pHNAH8I-1, the *tnfxB1*-*tmexCD1-toprJ1* cluster is located within transposon Tn*5393* as part of a 15,695-bp insertion that also includes two genes encoding site-specific integrases (*int*) and two genes of unknown function (*hp1* and *hp2*) (Fig. 5). GenBank searches identified several sequences closely related (>97% identity) to all or part of this region, as well as uninterrupted versions of flanking sequences (Fig. 5). Site-specific integrases can capture and cotransfer large DNA fragments (31, 32) and, thus, may have been responsible for the acquisition of *tnfxB1*-*tmexCD1-toprJ1* and its variants from the chromosomes of *Pseudomonas* species by plasmids; however, this requires further investigation. In p18-29-MDR (GenBank accession number MK262712.1, human, K. pneumoniae, China), insertion sequence IS*26* truncates the hp2 gene, while a second IS*26* is located downstream (Fig. 5), suggesting transfer of the gene cluster to this plasmid mediated by IS*26* (33).

### Prevalence of *tmexCD1-toprJ1* in human *Enterobacteriaceae* isolates and animal-associated samples.

**FIG 6:**
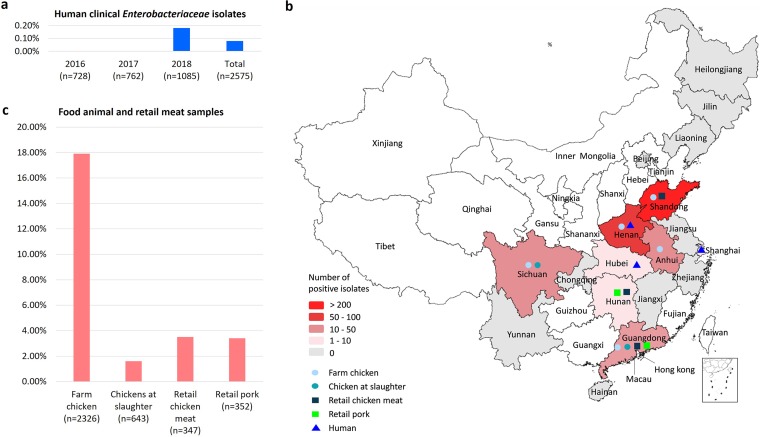
Prevalence and distribution of *tmexCD1-toprJ1*-positive isolates. (a) Prevalence of *tmexCD1* in human clinical *K. pneumonia* isolates, 2016 to 2018. (b) Geographical distribution of isolates harboring *tmexCD1-toprJ1* in China (collected between November 2018 and July 2019). Colors indicate the numbers of positive isolates and symbols indicate the sources of samples as shown in the key. Provinces that were not sampled are shown in white. The human *tmexCD1-toprJ1*-positive isolate from Henan Province was identified in GenBank (accession number MK262712.1). Map was generated by R version 3.5.2. (c) Prevalence of *tmexCD1*-*toprJ1*-positive isolates in food animal and retail meat samples from China, 2018 to 2019.

Of 2,575 K. pneumoniae isolates from various human clinical samples, only two (0.08%), recovered from gallbladder and lung samples in 2018, were positive for *tmexCD1-toprJ1* (Fig. 6a; Table S4). Both isolates (WH61 and ZS243) belonged to ST15, and they were collected in 2018 from hospitals located in Wuhan and Shanghai (Fig. 6b), respectively.

10.1128/mBio.02930-19.8 TABLE S4 Sites of infection by K. pneumoniae. Download Table S4, DOCX file, 0.02 MB. Copyright © 2020 Lv et al. 2020 Lv et al. This content is distributed under the terms of the Creative Commons Attribution 4.0 International license.

Among 2,326 cloacal swab samples from 25 chicken farms, 643 cecum samples from chickens at slaughter, 142 fecal samples from two pig farms, 347 chicken meat samples, and 352 retail pork samples, 17.9%, 1.6%, 0%, 3.5%, and 3.4%, respectively, contained *tmexCD1-toprJ1-*positive isolates (Fig. 6c; Table S5). Overall, 11.7% of the 3,810 samples contained *tmexCD1-toprJ1-*positive *Enterobacteriaceae* isolates, consisting of 415 K. pneumoniae, 26 Klebsiella oxytoca, and 6 Raoultella planticola isolates. Of the 25 chicken farms sampled in this study, 14 farms from five provinces produced samples contaminated with *tmexCD1-toprJ1*-positive *Enterobacteriaceae*, with detection rates varying from 2.0% to 100%. In addition, *tmexCD1-toprJ1*-positive isolates were detected in 8 of the 17 provinces sampled in this study (Fig. 6b).

10.1128/mBio.02930-19.9 TABLE S5 Prevalence of food animal and retail meat samples carrying *tmexCD1-toprJ1*-positive *Enterobacteriaceae* isolates. Download Table S5, DOCX file, 0.02 MB. Copyright © 2020 Lv et al. 2020 Lv et al. This content is distributed under the terms of the Creative Commons Attribution 4.0 International license.

## DISCUSSION

Here, we identified and characterized a novel plasmid-borne multidrug resistance gene cluster, designated *tmexCD1-toprJ1*, encoding proteins homologous to tripartite RND-type multidrug efflux systems. Based on its structure and function, we determined that TMexCD1-TOprJ1 is a new member of the MexCD-OprJ family of efflux pumps (34). TMexCD1-TOprJ1 confers resistance or reduced susceptibility to several classes of clinically important antimicrobials, including tetracyclines, glycylcycline (tigecycline), cephalosporins, aminoglycosides, phenicols, and also quinolones, and thus, could be considered a novel plasmid-mediated quinolone resistance (PMQR) determinant (35, 36). Of note, TMexCD1-TOprJ1 also confers resistance to eravacycline, a new synthetic analogue of the tetracycline family recently approved for clinical use, which has robust activity against MDR *Enterobacteriaceae*, especially CRE (37). More worryingly, the *tmexCD1-toprJ1* cluster not only confers resistance to multiple agents but is found on the same plasmid as and could be cotransferred with several other mobile resistance genes, including colistin resistance gene *mcr-8.1*. Thus, the acquisition of a *tmexCD1-toprJ1*-positive plasmid by *Enterobacteriaceae*, especially by CRE, has the potential to generate pandrug-resistant strains, such as AH8I, resulting in untreatable infections. In this context, the emergence of this novel transferable MDR efflux pump constitutes a particularly serious threat to antimicrobial therapy and is of significance for clinical management and public health.

MexCD-OprJ is one of the intrinsic, chromosomal, RND-type MDR pumps of *Pseudomonas* spp. (4). Previous studies demonstrated that the expression of MexCD-OprJ is regulated by NfxB and that it is usually not expressed in wild-type strains. Overproduction of MexCD-OprJ in *nfxB* loss-of-function mutants causes increased resistance to tetracyclines, fluoroquinolones, cephalosporins, macrolides, and chloramphenicol (4, 23, 34, 38). However, the resistance levels conferred by TMexCD1-TOprJ1 here were not affected by the presence of *tnfxB1* upstream from *tmexCD1-toprJ1*. In addition, like P. aeruginosa MexCD-OprJ, the expression of TMexCD1 alone in E. coli reduced the susceptibility of the recombinant strain to various antimicrobials, indicating that TMexCD1 might function in E. coli in conjunction with the chromosomally encoded outer membrane protein TolC (39). Further studies are needed to assess the contribution of individual components of TMexCD1-TOprJ1 to antibiotic resistance and substrate specificity and to clarify any interactions between TMexCD1 and other RND-type pumps inherent in *Enterobacteriaceae*.

TMexCD1-TOprJ1 is the first plasmid-borne RND-type tigecycline and eravacycline resistance determinant to be characterized. It is not surprising that TMexCD1-TOprJ1 mediates tigecycline resistance, given that tigecycline is a substrate for many other RND pumps in various species and that multidrug efflux pumps of P. aeruginosa are functional and retain their substrate specificity in E. coli (4, 39, 40). Although the expression of P. aeruginosa MexCD-OprJ results in reduced susceptibility to tigecycline, glycylcyclines are generally inferior substrates compared with narrower-spectrum tetracyclines (23). However, the increases in tigecycline and eravacycline MICs in the presence of TMexCD1-TOprJ1 were equal to or higher than those for narrower-spectrum tetracyclines. The change of substrate specificity could be due to substitution of key residues and structural changes compared to the sequences and structures of other RND pumps, such as AcrAB-TolC and MexCD-OprJ. Previous work showed that tetracycline was recognized at the bottom of the multisubstrate-binding site and mostly toward the distal pocket in AcrB of E. coli (41–43). There are several amino acid differences in these regions of TMexD1 compared with the sequences of MexD and AcrB, which might cause changes in the physicochemical properties of the substrate-binding pocket. However, further structural studies of TMexCD1-TOprJ1 are needed to elucidate the reasons for these changes in susceptibility.

*tmexCD1-toprJ1* appears to have originated from the chromosome of a *Pseudomonas* species and may have been transferred onto plasmids by site-specific integrases encoded by adjacent genes. In general, resistance genes in the chromosome of the source bacterium are captured and integrated into a plasmid, which can then be disseminated within and among bacterial species (44, 45). Such mobile resistance is often caused by the transfer of individual genes, and the transfer of an entire gene cluster encoding all three components of a tripartite drug efflux pump might be expected to be rarer (4). Although *Enterobacteriaceae* producing TMexCD1-TOprJ1 itself have not yet been detected outside China, the findings shown in Fig. 3 and 5 illustrate that related plasmid-encoded RND-type pumps are already present on several continents (Asia, Africa, Europe, and North America) (6, 24). Other less closely related plasmid-borne gene clusters encoding RND-type tripartite drug efflux pumps have also been reported, including on pB4 (uncultured bacterium, Germany) (6) and pNDM-CIT (Citrobacter freundii, India) (27). The increasing discovery of RND pump genes on plasmids is quite surprising but may suggest that their capture is more common than previously suspected and that chromosomally located genes encoding multidrug resistance efflux pumps in genera such as *Pseudomonas* might be an important reservoir of resistance genes for *Enterobacteriaceae*. Therefore, the identification of the first plasmid-encoded RND-type tigecycline resistance determinant, TMexCD1-TOprJ1, has global significance.

We identified *tmexCD1-toprJ1* in isolates from different sources, including humans, animals, and animal food, in multiple regions of China (Fig. 6b), indicating that it has disseminated widely. The earliest reported *tmexCD1-toprJ1*-positive isolate that we identified from GenBank searches was collected in 2014 from a chicken in Shandong Province, China (GenBank accession number QFMD01000298), suggesting that this cluster began to emerge on plasmids at least 4 years ago. However, *tmexCD1-toprJ1* still appears to be rare in human clinical K. pneumoniae isolates in China (<0.1%) and, to date, has only been detected in three isolates, collected in 2018, two identified in this study plus one other, the sequence of which is available under GenBank accession number MK262712.1. Thus, like *mcr-1*, *tmexCD1-toprJ1* may have emerged in livestock-derived bacterial strains (46) and is only now beginning to make its way to humans. However, considering the apparent transferability of the *tmexCD1-toprJ1* gene cluster and the broad substrate spectrum of TMexCD1-TOprJ1, further dissemination of this plasmid-borne tigecycline resistance gene cluster is possible.

Plasmids carrying *tmexCD1-toprJ1* are so far limited to K. pneumoniae and the closely related species K. oxytoca, R. planticola, and Raoultella ornithinolytica (GenBank accession number QWIX01000043.1). This may be because TMexCD1-TOprJ1 imposes a fitness cost (Fig. 2) and/or the plasmids carrying *tmexCD1-toprJ1* have a narrow host range and/or are not stable in other *Enterobacteriaceae* species, such as E. coli (Fig. 4b). However, IS*26* may have been responsible for transferring *tmexCD1-toprJ1* (Fig. 5). IS*26*-mediated transposition is most effective when targeting existing copies of itself (33) and IS*26* is already common in multidrug resistance plasmids, so further IS*26*-mediated mobilization of *tmexCD1-toprJ1* among different plasmid types is possible. This may lead to the rapid and widespread dispersion of *tmexCD1-toprJ1* in *Enterobacteriaceae*.

Tigecycline has not been used in veterinary clinics; however, selective pressure imposed by heavy use of older tetracyclines (doxycycline, chlortetracycline, and oxytetracycline) and other antimicrobials (enrofloxacin, florfenicol, and cephalosporins) in China could have contributed to the emergence of this multisubstrate efflux pump (47). The broad substrate spectrum might also explain why the prevalence rates for TMexCD1-TOprJ1-producing *Enterobacteriaceae* in animal-associated samples are higher than the rates reported for *tet*(X3) and *tet*(X4), which specifically confer tetracycline resistance (20, 21).

In conclusion, we report the emergence of a novel plasmid-mediated multidrug resistance RND family efflux pump, TMexCD1-TOprJ1, which is capable of conferring resistance to tetracyclines, including tigecycline and eravacycline, and reduced susceptibility to many other clinically important antimicrobial agents in *Enterobacteriaceae*, constituting a serious emerging resistance problem. In particular, *tmexCD1-toprJ1* is widespread among K. pneumoniae isolates from food animals, and this is one of the most problematic pathogens in human clinical settings. *tmexCD1-toprJ1* and its variants were likely transferred from the chromosomes of *Pseudomonas* spp. onto plasmids that are already present in strains from multiple continents (Asia, Europe, and North America). Further global dissemination of these transferable MDR efflux pumps among *Enterobacteriaceae* is likely, presenting a new and severe challenge for clinical management. The current low prevalence of *tmexCD1-toprJ1* in human clinical isolates provides a precious time window to design and implement control measures. Taking a “One Health” perspective, measures to monitor and control the spread of plasmid-borne *mexCD-oprJ*-like gene clusters to other bacterial species, including clinical strains, are urgently needed.

## MATERIALS AND METHODS

### Bacterial isolates.

As part of a project (National Natural Science Foundation of China, grant no. 81661138002) investigating CRE carriage by food-producing animals in China in May 2017, we collected 43 cloacal swab samples from one chicken farm located in eastern China. Samples were precultured in LB broth for 18 h and then inoculated onto MacConkey agar plates with 1 mg/liter imipenem for the selection of CRE isolates. Colonies with different morphologies were selected for PCR screening of carbapenemase genes. Species identification was performed by matrix-assisted laser desorption ionization–time of flight mass spectrometry (MALDI-TOF MS) (Shimadzu Corp., Kyoto, Japan). A total of 25 CRE isolates, including 9 E. coli, 8 K. pneumoniae, and 8 other *Enterobacteriaceae* isolates, were obtained from 25 samples. Antimicrobial susceptibility testing results showed that five K. pneumoniae isolates (AH6I, AH8I, AH25I, AH28I, and AH33I) exhibited resistance to tigecycline (MIC = 16 mg/liter) (Table 1). Clonal relatedness of the five K. pneumoniae isolates was determined by pulsed-field gel electrophoresis (PFGE) and multilocus sequence typing (MLST) (https://bigsdb.pasteur.fr/klebsiella/klebsiella.html ). Strains used for conjugation, transformation, and stability studies are listed in Table S2 in the supplemental material. MICs of antibiotics against original isolates, transconjugants, and transformants were determined by agar dilution or broth microdilution (tigecycline and colistin) according to the CLSI guidelines (53). Tigecycline MICs were also measured in the presence of the efflux pump inhibitor 1-(1-naphthylmethyl)-piperazine (NMP) (final concentration, 75 mg/liter).

### Whole-genome sequencing and annotation.

K. pneumoniae isolate AH8I carrying *tmexCD1*-*toprJ1* was subjected to whole-genome sequencing (WGS) using both short-read (Illumina, San Diego, CA, USA) and long-read (PacBio, Menlo Park, CA, USA) platforms. PacBio reads and Illumina reads were combined to produce a *de novo* hybrid assembly using Unicycler version 0.4.3 (48). Multilocus sequence types (STs), antimicrobial resistance genes, and plasmid replicon types were identified using the Bacterial Analysis Pipeline from GoSeqIt (https://www.goseqit.com/). ISfinder (https://www-is.biotoul.fr/) and Galileo AMR (https://galileoamr.arcbio.com/mara/; Arc Bio, Cambridge, MA), formerly the Multiple Antibiotic Resistance Annotator (MARA) (49), were used to analyze the multidrug resistance regions and plasmids.

### Gene cloning and bacterial growth.

DNA fragments containing *nfxB1*-*tmexCD1*-*toprJ1*, *tmexCD1*-*toprJ1*, *nfxB1*-*tmexCD1*, or *tmexCD1* were amplified using primers listed in Table S6 in the supplemental material and ligated into pHSG575, yielding pHSG575-tnfxB1-tmexCD1-toprJ1, pHSG575-tmexCD1-toprJ1, pHSG575-tnfxB1-tmexCD1, and pHSG575-tmexCD1. The four recombinant plasmids were then transformed into electrocompetent E. coli DH5α cells. pHSG575-tnfxB1-tmexCD1-toprJ1 was also transferred into K. pneumoniae isolate YX94 and *S.* Typhimurium isolate HN227.Transformants were selected on MacConkey agar plates containing 4 mg/liter tigecycline and 30 mg/liter chloramphenicol. E. coli DH5α, K. pneumoniae YX94, and *S.* Typhimurium HN227 with or without pHSG575 or pHSG575-tnfxB1-tmexCD1-toprJ1 were inoculated into LB broth and shaken at 220 rpm at 37°C. The overnight cultures were diluted 1:1,000 (100 μl/100 ml) in fresh LB broth. Bacterial suspensions were cultured at 37°C in triplicate and shaken at 220 rpm at 37°C for 12 h. Bacterial growth was monitored by measuring the optical density at 600 nm (OD_600_) on a Multiskan spectrum microplate spectrophotometer (Thermo Labsystems, Frankin, MA) at hourly intervals. Growth curves were generated with GraphPad Prism version 6.0 (GraphPad Software, Inc., La Jolla, CA, USA).

10.1128/mBio.02930-19.10 TABLE S6 Primers used in this study. Download Table S6, DOCX file, 0.02 MB. Copyright © 2020 Lv et al. 2020 Lv et al. This content is distributed under the terms of the Creative Commons Attribution 4.0 International license.

### Plasmid transferability and stability.

The transferability and transfer frequency of tigecycline resistance was investigated by conjugation experiments using azide-resistant E. coli strain J53 and carbapenem-resistant K. pneumoniae AH58I as recipients. Transconjugants were selected on MacConkey agar plates supplemented with tigecycline (4 mg/liter) or colistin (4 mg/liter) plus azide (200 mg/liter) or imipenem (2 mg/liter). Transfer frequencies were calculated as the number of transconjugants obtained per recipient.

Plasmid pHNAH8I-1 was extracted from K. pneumoniae AH8I and used to transform tigecycline-susceptible strains belonging to different species, namely, K. pneumoniae YX94, *S.* Typhimurium HN227, E. coli DH5α, and azide-resistant E. coli J53, by electroporation. Transformants were selected on MacConkey agar plates containing 4 mg/liter tigecycline.

The stability of pHNAH8I-1 carrying *tmexCD1-toprJ1* in AH8I and transconjugants (E. coli J53/pHNAH8I-1 and K. pneumoniae AH58I/pHNAH8I-1) was studied by passage in antibiotic-free Luria broth (LB). Three separate cultures of each strain carrying the plasmid were grown in 3 ml antibiotic-free LB overnight at 37°C, followed by serial passage of 3 μl overnight culture into 3 ml LB each day, yielding 10 generations for each strain, lasting 15 days. Every 3 days, samples were collected and streaked on antibiotic-free MacConkey agar plates. One hundred colonies were selected, and the presence of *tmexCD1-toprJ1* and pHNAH8I-1 was confirmed by PCR amplification of *tmexCD1* and *repA*, respectively, using primers listed in Table S6 in the supplemental material. Plasmid retention was calculated as the percentage of cells with both *tmexCD1* and *repA* in the 100 selected colonies.

### Tigecycline accumulation assay.

Tigecycline accumulation was assessed as described by Mortimer and Piddock (50), with several modifications. E. coli DH5α/pHSG575 or E. coli DH5α/pHSG575-tmexCD1 was cultured in LB broth at 37°C overnight with shaking. Cultures were diluted 100-fold in fresh LB and then cultured at 37°C with shaking to late logarithmic phase. Cells were then harvested by centrifugation at 4°C, washed twice in 50 mM phosphate-buffered saline (PBS; pH 7.0), and resuspended in the same buffer to an optical density at 600 nm of 1.0. The cell suspension was incubated for 15 min at 37°C, and 500-μl samples were collected for use as matrix blanks for the drug accumulation measurements. Tigecycline was then added to the suspension at a final concentration of 100 mg/liter. Three 500-μl samples were collected at various time points (10, 30, 40, 50, and 60 min postinoculation), with an equivalent volume of cells also collected to measure the bacterial dry weight at each time point. Following incubation for 30 min, NMP was added to one half of the suspension (final concentration, 100 mg/liter), while the other half was used as a control without NMP. Each sample was immediately diluted with 500 μl of ice-cold PBS and centrifuged at 6,000 rpm for 10 min at 4°C. Bacterial cell pellets were washed once with 1 ml of ice-cold PBS and then resuspended in 1 ml of 0.1 M glycine hydrochloride (pH 3.0) and shaken at room temperature for 3 h. Samples were then centrifuged at 12,000 rpm for 10 min, and the resulting supernatants were filtered through 0.2-μm pore diameter filters. Finally, the concentration of tigecycline in the supernatant was analyzed using high-performance liquid chromatography apparatus (Shimadzu Corp.) equipped with a diode array detector (51) at a detection wavelength of 244 nm. Data are shown as micrograms of tigecycline accumulated per gram dry bacterial weight.

### *In vivo* study of the contribution of TMexCD1-TOprJ1 to tigecycline resistance.

The animal study was approved by the institutional Animal Ethics Committee [approval number SYXK (YUE)2019-0136], and animals were maintained in accordance with the SCAU Laboratory Animal Welfare and Animal Experimental Ethical Inspection, as issued by the SCAU Committee on Animal Welfare and Experimental Ethics (approval number 2019075). Female specific-pathogen-free BALB/c mice weighing 18 to 22 g were obtained from Southern Medical University (Guangzhou, China) and used in a neutropenic-mouse thigh infection model. Prior to mouse thigh inoculation, mice were acclimated for 5 days in the Laboratory Animal Center of South China Agricultural University. Mice were randomly divided into four groups, with 6 mice per group. Neutropenia was induced by continuous intraperitoneal injection of cyclophosphamide for 4 days (150 mg/kg of body weight) and then at 1 day (100 mg/kg) prior to thigh infection, as previously described (52). Mice were infected by intramuscular injection of a 50-μl suspension (3 × 10^6^ CFU/milliliter) of K. pneumoniae AH58I (susceptible to tigecycline) or K. pneumoniae AH58I/pHNAH8I-1 into the left thigh muscle. In the treatment group, mice were treated every 12 h with intraperitoneal injections of tigecycline (12 mg/kg), while the same dose of saline was injected every 12 h in the nontreatment group. Mice were sacrificed 36 h after the first administration, and thigh muscles were removed aseptically and homogenized in 2 ml of saline solution, followed by further homogenization in 3 ml of saline. To evaluate the concentrations of K. pneumoniae bacteria in the initial inocula, 50-μl aliquots of 10-fold serial dilutions of the bacterial suspensions were plated on LB agar plates.

### Screening additional isolates from China carrying *tmexCD1-toprJ1*.

We examined 2,575 clinical K. pneumoniae isolates collected between 2016 and 2018 at 18 tertiary hospitals from 15 provinces in China. To determine the occurrence of *tmexCD1*-*toprJ1* in *Enterobacteriaceae* from farm animals, we collected 2,326 cloacal swab samples from 25 chicken farms located in 10 provinces, 142 rectal swab samples from two pig farms in 1 province, 643 cecum samples from chickens at slaughter from 5 provinces, and 699 retail meat samples (347 chicken samples and 352 pork samples) from farmers’ markets and supermarkets in 3 provinces in China between November 2018 and July 2019 (Table S5 in the supplemental material). Samples were inoculated onto MacConkey agar plates containing 2 mg/liter tigecycline. Fifteen human clinical K. pneumoniae isolates showing reduced susceptibility to tigecycline (MICs of >1 mg/liter) by broth microdilution, along with *Enterobacteriaceae* isolates collected from food animals (*n* = 3,111) and meat samples (*n* = 699) that grew in the presence of 2 mg/liter tigecycline (Table S5), were screened for the presence of *tmexCD1*-*toprJ1* by PCR using primers amplifying part of the *tmexC1* and *tmexD1* sequences (Table S6). PCR amplicons were sequenced using Sanger’s method.

### Data availability.

The nucleotide sequence of plasmid pHNAH8I-1 was deposited in the GenBank database under accession number MK347425.

10.1128/mBio.02930-19.3 FIG S3 Amino acid sequence alignments and predicted secondary structures of TMexD1 from Klebsiella pneumoniae AH8I and homologs. Alignments were generated with ClustalX 2 software then visualized using the ESPript server (http://espript.ibcp.fr/ESPript/cgi-bin/ESPript.cgi). Identical and similar residues are boxed in red and blue, respectively. Secondary structure is shown on the lines above the sequence alignment with α-helices marked by coils, β-strands by arrows, and strict β-turns by TT or TTT. The PDB IDs are as follows: NP_249117.1 (MexB); NP_253288.1 (MexD); NP_414995.1 (AcrB). The last letter (P or C) of each protein name indicates the location of the coding sequence (P, encoded on a plasmid; C, encoded on the chromosome). Download FIG S3, TIF file, 2.6 MB. Copyright © 2020 Lv et al. 2020 Lv et al. This content is distributed under the terms of the Creative Commons Attribution 4.0 International license.

10.1128/mBio.02930-19.4 FIG S4 Amino acid sequence alignments and predicted secondary structures of TOprJ1 from Klebsiella pneumoniae AH8I and homologs. Alignments were generated with ClustalX 2 software then visualized using the ESPript server (http://espript.ibcp.fr/ESPript/cgi-bin/ESPript.cgi). Identical and similar residues are boxed in red and blue, respectively. Secondary structure is shown on the lines above the sequence alignment with α-helices marked by coils, β-strands by arrows, and strict β-turns by TT or TTT. The PDB IDs are as follows: NP_249118.1 (OprM); NP_253287.1 (OprJ); NP_417507.2 (TolC). The last letter (P or C) of each protein name indicates the coding sequence location (P, encoded on a plasmid; C, encoded on the chromosome). Download FIG S4, TIF file, 1.8 MB. Copyright © 2020 Lv et al. 2020 Lv et al. This content is distributed under the terms of the Creative Commons Attribution 4.0 International license.

## References

[1] Tacconelli E, Carrara E, Savoldi A, Harbarth S, Mendelson M, Monnet DL, Pulcini C, Kahlmeter G, Kluytmans J, Carmeli Y, Ouellette M, Outterson K, Patel J, Cavaleri M, Cox EM, Houchens CR, Grayson ML, Hansen P, Singh N, Theuretzbacher U, Magrini N, WHO Pathogens Priority List Working Group. 2018 Discovery, research, and development of new antibiotics: the WHO priority list of antibiotic-resistant bacteria and tuberculosis. Lancet Infect Dis 18:318–327. doi:10.1016/S1473-3099(17)30753-3. 29276051

[2] Navon-Venezia S, Kondratyeva K, Carattoli A 2017 *Klebsiella pneumoniae*: a major worldwide source and shuttle for antibiotic resistance. FEMS Microbiol Rev 41:252–275. doi:10.1093/femsre/fux013. 28521338

[3] Carattoli A 2013 Plasmids and the spread of resistance. Int J Med Microbiol 303:298–304. doi:10.1016/j.ijmm.2013.02.001. 23499304

[4] Li XZ, Plésiat P, Nikaido H 2015 The challenge of efflux-mediated antibiotic resistance in Gram-negative bacteria. Clin Microbiol Rev 28:337–418. doi:10.1128/CMR.00117-14. 25788514 PMC4402952

[5] Du D, Wang-Kan X, Neuberger A, van Veen HW, Pos KM, Piddock LJV, Luisi BF 2018 Multidrug efflux pumps: structure, function and regulation. Nat Rev Microbiol 16:523–539. doi:10.1038/s41579-018-0048-6. 30002505

[6] Tauch A, Schlüter A, Bischoff N, Goesmann A, Meyer F, Pühler A 2003 The 79,370-bp conjugative plasmid pB4 consists of an IncP-1β backbone loaded with a chromate resistance transposon, the *strA*-*strB* streptomycin resistance gene pair, the oxacillinase gene *bla*_NPS-1_, and a tripartite antibiotic efflux system of the resistance-nodulation-division family. Mol Genet Genomics 268:570–584. doi:10.1007/s00438-002-0785-z. 12589432

[7] Sheu CC, Chang YT, Lin SY, Chen YH, Hsueh PR 2019 Infections caused by carbapenem-resistant Enterobacteriaceae: an update on therapeutic options. Front Microbiol 10:80. doi:10.3389/fmicb.2019.00080. 30761114 PMC6363665

[8] Liu Y-Y, Wang Y, Walsh TR, Yi L-X, Zhang R, Spencer J, Doi Y, Tian G, Dong B, Huang X, Yu L-F, Gu D, Ren H, Chen X, Lv L, He D, Zhou H, Liang Z, Liu J-H, Shen J 2016 Emergence of plasmid-mediated colistin resistance mechanism MCR-1 in animals and human beings in China: a microbiological and molecular biological study. Lancet Infect Dis 16:161–168. doi:10.1016/S1473-3099(15)00424-7. 26603172

[9] Grossman TH 2016 Tetracycline antibiotics and resistance. Cold Spring Harb Perspect Med 6:a025387. doi:10.1101/cshperspect.a025387. 26989065 PMC4817740

[10] Wang Q, Wang X, Wang J, Ouyang P, Jin C, Wang R, Zhang Y, Jin L, Chen H, Wang Z, Zhang F, Cao B, Xie L, Liao K, Gu B, Yang C, Liu Z, Ma X, Jin L, Zhang X, Man S, Li W, Pei F, Xu X, Jin Y, Ji P, Wang H 2018 Phenotypic and genotypic characterization of carbapenem-resistant Enterobacteriaceae: data from a longitudinal large-scale CRE study in China (2012–2016). Clin Infect Dis 67:S196–S205. doi:10.1093/cid/ciy660. 30423057

[11] Pitout JD, Nordmann P, Poirel L 2015 Carbapenemase-producing *Klebsiella pneumoniae*, a key pathogen set for global nosocomial dominance. Antimicrob Agents Chemother 59:5873–5884. doi:10.1128/AAC.01019-15. 26169401 PMC4576115

[12] David S, Reuter S, Harris SR, Glasner C, Feltwell T, Argimon S, Abudahab K, Goater R, Giani T, Errico G, Aspbury M, Sjunnebo S, EuSCAPE Working Group, ESGEM Study Group, Feil EJ, Rossolini GM, Aanensen DM, Grundmann H 2019 Epidemic of carbapenem-resistant *Klebsiella pneumoniae* in Europe is driven by nosocomial spread. Nat Microbiol 4:1919–1929. doi:10.1038/s41564-019-0492-8. 31358985 PMC7244338

[13] Xu L, Sun X, Ma X 2017 Systematic review and meta-analysis of mortality of patients infected with carbapenem-resistant *Klebsiella pneumoniae*. Ann Clin Microbiol Antimicrob 16:18. doi:10.1186/s12941-017-0191-3. 28356109 PMC5371217

[14] Sheng ZK, Hu F, Wang W, Guo Q, Chen Z, Xu X, Zhu D, Wang M 2014 Mechanisms of tigecycline resistance among *Klebsiella pneumoniae* clinical isolates. Antimicrob Agents Chemother 58:6982–6985. doi:10.1128/AAC.03808-14. 25182649 PMC4249433

[15] Bialek-Davenet S, Lavigne JP, Guyot K, Mayer N, Tournebize R, Brisse S, Leflon-Guibout V, Nicolas-Chanoine MH 2015 Differential contribution of AcrAB and OqxAB efflux pumps to multidrug resistance and virulence in Klebsiella pneumoniae. J Antimicrob Chemother 70:81–88. doi:10.1093/jac/dku340. 25193085

[16] Wang X, Chen H, Zhang Y, Wang Q, Zhao C, Li H, He W, Zhang F, Wang Z, Li S, Wang H 2015 Genetic characterisation of clinical *Klebsiella pneumoniae* isolates with reduced susceptibility to tigecycline: role of the global regulator RamA and its local repressor RamR. Int J Antimicrob Agents 45:635–640. doi:10.1016/j.ijantimicag.2014.12.022. 25681067

[17] Chen Y, Hu D, Zhang Q, Liao XP, Liu YH, Sun J 2017 Efflux pump overexpression contributes to tigecycline heteroresistance in *Salmonella enterica serovar* Typhimurium. Front Cell Infect Microbiol 7:37. doi:10.3389/fcimb.2017.00037. 28261566 PMC5313504

[18] He F, Shi Q, Fu Y, Xu J, Yu Y, Du X 2018 Tigecycline resistance caused by *rpsJ* evolution in a 59-year-old male patient infected with KPC-producing *Klebsiella pneumoniae* during tigecycline treatment. Infect Genet Evol 66:188–191. doi:10.1016/j.meegid.2018.09.025. 30268919

[19] Yao H, Qin S, Chen S, Shen J, Du XD 2018 Emergence of carbapenem-resistant hypervirulent *Klebsiella pneumoniae*. Lancet Infect Dis 18:25. doi:10.1016/S1473-3099(17)30628-X. 29102518

[20] He T, Wang R, Liu D, Walsh TR, Zhang R, Lv Y, Ke Y, Ji Q, Wei R, Liu Z, Shen Y, Wang G, Sun L, Lei L, Lv Z, Li Y, Pang M, Wang L, Sun Q, Fu Y, Song H, Hao Y, Shen Z, Wang S, Chen G, Wu C, Shen J, Wang Y 2019 Emergence of plasmid-mediated high-level tigecycline resistance genes in animals and humans. Nat Microbiol 4:1450–1456. doi:10.1038/s41564-019-0445-2. 31133751

[21] Sun J, Chen C, Cui CY, Zhang Y, Liu X, Cui ZH, Ma XY, Feng Y, Fang LX, Lian XL, Zhang RM, Tang YZ, Zhang KX, Liu HM, Zhuang ZH, Zhou SD, Lv JN, Du H, Huang B, Yu FY, Mathema B, Kreiswirth BN, Liao XP, Chen L, Liu YH 2019 Plasmid-encoded *tet*(X) genes that confer high-level tigecycline resistance in *Escherichia coli*. Nat Microbiol 4:1457–1464. doi:10.1038/s41564-019-0496-4. 31235960 PMC6707864

[22] Wang L, Liu D, Lv Y, Cui L, Li Y, Li T, Song H, Hao Y, Shen J, Wang Y, Walsh TR 2019 Novel plasmid-mediated *tet*(X5) gene conferring resistance to tigecycline, eravacycline and omadacycline in clinical *Acinetobacter baumannii*. Antimicrob Agents Chemother 64:e01326-19. doi:10.1128/AAC.01326-19. 31611352 PMC7187588

[23] Dean CR, Visalli MA, Projan SJ, Sum PE, Bradford PA 2003 Efflux-mediated resistance to tigecycline (GAR-936) in *Pseudomonas aeruginosa* PAO1. Antimicrob Agents Chemother 47:972–978. doi:10.1128/aac.47.3.972-978.2003. 12604529 PMC149306

[24] Flach CF, Johnning A, Nilsson I, Smalla K, Kristiansson E, Larsson DG 2015 Isolation of novel IncA/C and IncN fluoroquinolone resistance plasmids from an antibiotic-polluted lake. J Antimicrob Chemother 70:2709–2717. doi:10.1093/jac/dkv167. 26124213

[25] Zheng B, Huang C, Xu H, Yu X, Zhang J, Wang X, Jiang X, Xiao Y, Li L 2018 Complete nucleotide sequences of two KPC-2-encoding plasmids from the same *Citrobacter freundii* isolate. J Antimicrob Chemother 73:531–533. doi:10.1093/jac/dkx381. 29092035

[26] Yuan M, Chen H, Zhu X, Feng J, Zhan Z, Zhang D, Chen X, Zhao X, Lu J, Xu J, Zhou D, Li J 2017 pSY153-MDR, a p12969-DIM-related mega plasmid carrying *bla*_IMP-45_ and *armA*, from clinical *Pseudomonas putida*. Oncotarget 8:68439–68447. doi:10.18632/oncotarget.19496. 28978128 PMC5620268

[27] Dolejska M, Villa L, Poirel L, Nordmann P, Carattoli A 2013 Complete sequencing of an IncHI1 plasmid encoding the carbapenemase NDM-1, the ArmA 16S RNA methylase and a resistance-nodulation-cell division/multidrug efflux pump. J Antimicrob Chemother 68:34–39. doi:10.1093/jac/dks357. 22969080

[28] Stover CK, Pham XQ, Erwin AL, Mizoguchi SD, Warrener P, Hickey MJ, Brinkman FS, Hufnagle WO, Kowalik DJ, Lagrou M, Garber RL, Goltry L, Tolentino E, Westbrock-Wadman S, Yuan Y, Brody LL, Coulter SN, Folger KR, Kas A, Larbig K, Lim R, Smith K, Spencer D, Wong GK, Wu Z, Paulsen IT, Reizer J, Saier MH, Hancock RE, Lory S, Olson MV 2000 Complete genome sequence of *Pseudomonas aeruginosa* PAO1, an opportunistic pathogen. Nature 406:959–964. doi:10.1038/35023079. 10984043

[29] Blattner FR, Plunkett G III, Bloch CA, Perna NT, Burland V, Riley M, Collado-Vides J, Glasner JD, Rode CK, Mayhew GF, Gregor J, Davis NW, Kirkpatrick HA, Goeden MA, Rose DJ, Mau B, Shao Y 1997 The complete genome sequence of *Escherichia coli* K-12. Science 277:1453–1462. doi:10.1126/science.277.5331.1453. 9278503

[30] Nolivos S, Cayron J, Dedieu A, Page A, Delolme F, Lesterlin C 2019 Role of AcrAB-TolC multidrug efflux pump in drug-resistance acquisition by plasmid transfer. Science 364:778–782. doi:10.1126/science.aav6390. 31123134

[31] Ravatn R, Studer S, Zehnder AJ, van der Meer JR 1998 Int-B13, an unusual site-specific recombinase of the bacteriophage P4 integrase family, is responsible for chromosomal insertion of the 105-kilobase clc element of *Pseudomonas* sp. strain B13. J Bacteriol 180:5505–5514. doi:10.1128/JB.180.21.5505-5514.1998. 9791097 PMC107606

[32] Groth AC, Calos MP 2004 Phage integrases: biology and applications. J Mol Biol 335:667–678. doi:10.1016/j.jmb.2003.09.082. 14687564

[33] Harmer CJ, Moran RA, Hall RM 2014 Movement of IS*26*-associated antibiotic resistance genes occurs via a translocatable unit that includes a single IS*26* and preferentially inserts adjacent to another IS*26*. mBio 5:e01801-14. doi:10.1128/mBio.01801-14. 25293759 PMC4196232

[34] Poole K, Gotoh N, Tsujimoto H, Zhao Q, Wada A, Yamasaki T, Neshat S, Yamagishi J, Li XZ, Nishino T 1996 Overexpression of the *mexC*-*mexD*-*oprJ* efflux operon in *nfxB*-type multidrug-resistant strains of *Pseudomonas aeruginosa*. Mol Microbiol 21:713–724. doi:10.1046/j.1365-2958.1996.281397.x. 8878035

[35] Jacoby GA, Strahilevitz J, Hooper DC 2014 Plasmid-mediated quinolone resistance. Microbiol Spectr 2:PLAS-0006-2013. doi:10.1128/microbiolspec.PLAS-0006-2013. PMC4288778 25584197

[36] Rodríguez-Martínez JM, Machuca J, Cano ME, Calvo J, Martínez-Martínez L, Pascual A 2016 Plasmid-mediated quinolone resistance: two decades on. Drug Resist Updat 29:13–29. doi:10.1016/j.drup.2016.09.001. 27912841

[37] Chakradhar S 2016 What’s old is new: reconfiguring known antibiotics to fight drug resistance. Nat Med 22:1197–1199. doi:10.1038/nm1116-1197. 27824813

[38] Masuda N, Gotoh N, Ohya S, Nishino T 1996 Quantitative correlation between susceptibility and OprJ production in NfxB mutants of *Pseudomonas aeruginosa*. Antimicrob Agents Chemother 40:909–913. doi:10.1128/AAC.40.4.909. 8849250 PMC163229

[39] Srikumar R, Kon T, Gotoh N, Poole K 1998 Expression of *Pseudomonas aeruginosa* multidrug efflux pumps MexA-MexB-OprM and MexC-MexD-OprJ in a multidrug-sensitive *Escherichia coli* strain. Antimicrob Agents Chemother 42:65–71. doi:10.1128/AAC.42.1.65. 9449262 PMC105457

[40] Hirata T, Saito A, Nishino K, Tamura N, Yamaguchi A 2004 Effects of efflux transporter genes on susceptibility of *Escherichia coli* to tigecycline (GAR-936). Antimicrob Agents Chemother 48:2179–2184. doi:10.1128/AAC.48.6.2179-2184.2004. 15155219 PMC415592

[41] Murakami S, Nakashima R, Yamashita E, Matsumoto T, Yamaguchi A 2006 Crystal structures of a multidrug transporter reveal a functionally rotating mechanism. Nature 443:173–179. doi:10.1038/nature05076. 16915237

[42] Vargiu AV, Nikaido H 2012 Multidrug binding properties of the AcrB efflux pump characterized by molecular dynamics simulations. Proc Natl Acad Sci U S A 109:20637–20642. doi:10.1073/pnas.1218348109. 23175790 PMC3528587

[43] Jamshidi S, Sutton JM, Rahman KM 2018 Mapping the dynamic functions and structural features of AcrB efflux pump transporter using accelerated molecular dynamics simulations. Sci Rep 8:10470. doi:10.1038/s41598-018-28531-6. 29992991 PMC6041327

[44] Peterson E, Kaur P 2018 Antibiotic resistance mechanisms in bacteria: relationships between resistance determinants of antibiotic producers, environmental bacteria, and clinical pathogens. Front Microbiol 9:2928. doi:10.3389/fmicb.2018.02928. 30555448 PMC6283892

[45] Wellington EM, Boxall AB, Cross P, Feil EJ, Gaze WH, Hawkey PM, Johnson-Rollings AS, Jones DL, Lee NM, Otten W, Thomas CM, Williams AP 2013 The role of the natural environment in the emergence of antibiotic resistance in gram-negative bacteria. Lancet Infect Dis 13:155–165. doi:10.1016/S1473-3099(12)70317-1. 23347633

[46] Liu Y, Liu JH 2018 Monitoring colistin resistance in food animals, an urgent threat. Expert Rev Anti Infect Ther 16:443–446. doi:10.1080/14787210.2018.1481749. 29865881

[47] Van Boeckel TP, Brower C, Gilbert M, Grenfell BT, Levin SA, Robinson TP, Teillant A, Laxminarayan R 2015 Global trends in antimicrobial use in food animals. Proc Natl Acad Sci U S A 112:5649–5654. doi:10.1073/pnas.1503141112. 25792457 PMC4426470

[48] Wick RR, Judd LM, Gorrie CL, Holt KE 2017 Unicycler: resolving bacterial genome assemblies from short and long sequencing reads. PLoS Comput Biol 13:e1005595. doi:10.1371/journal.pcbi.1005595. 28594827 PMC5481147

[49] Partridge SR, Tsafnat G 2018 Automated annotation of mobile antibiotic resistance in Gram-negative bacteria: the Multiple Antibiotic Resistance Annotator (MARA) and database. J Antimicrob Chemother 73:883–890. doi:10.1093/jac/dkx513. 29373760

[50] Mortimer PG, Piddock LJ 1991 A comparison of methods used for measuring the accumulation of quinolones by Enterobacteriaceae, *Pseudomonas aeruginosa* and *Staphylococcus aureus*. J Antimicrob Chemother 28:639–653. doi:10.1093/jac/28.5.639. 1663928

[51] Li C, Sutherland CA, Nightingale CH, Nicolau DP 2004 Quantitation of tigecycline, a novel glycyclcycline, by liquid chromatography. J Chromatogr B Analyt Technol Biomed Life Sci 811:225–229. doi:10.1016/j.jchromb.2004.09.011. 15522724

[52] Lepak AJ, Zhao M, VanScoy B, Taylor DS, Ellis-Grosse E, Ambrose PG, Andes DR 2017 In vivo pharmacokinetics and pharmacodynamics of ZTI-01 (fosfomycin for injection) in the neutropenic murine thigh infection model against *Escherichia coli*, *Klebsiella pneumoniae*, and *Pseudomonas aeruginosa*. Antimicrob Agents Chemother 61:e00476-17. doi:10.1128/AAC.00476-17. 28396549 PMC5444146

[53] Clinical and Laboratory Standards Institute. 2017 Performance standards for antimicrobial susceptibility testing, 27th ed. Clinical and Laboratory Standards Institute, Wayne, PA.

