# Supplementary figures and images for: Emergence of a Plasmid-Encoded Resistance-Nodulation-Division Efflux Pump Conferring Resistance to Multiple Drugs, Including Tigecycline, in Klebsiella pneumoniae
Source: mBio. 2020 Mar 3;11(2):e02930-19. doi: 10.1128/mBio.02930-19 (PMC7064769; doi:10.1128/mBio.02930-19)

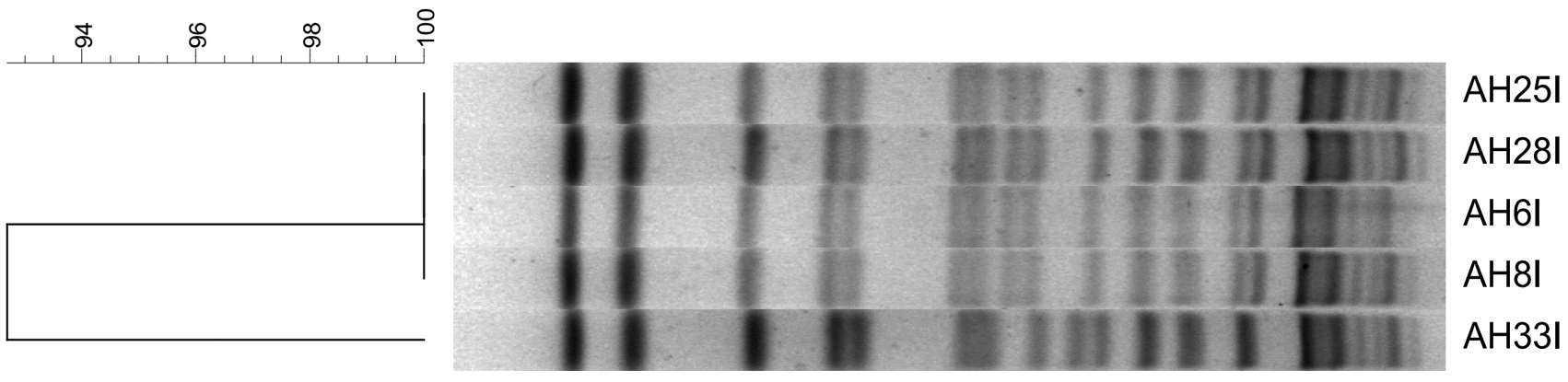

Supplement: FIG S1 [file mBio.02930-19-sf001.tif]

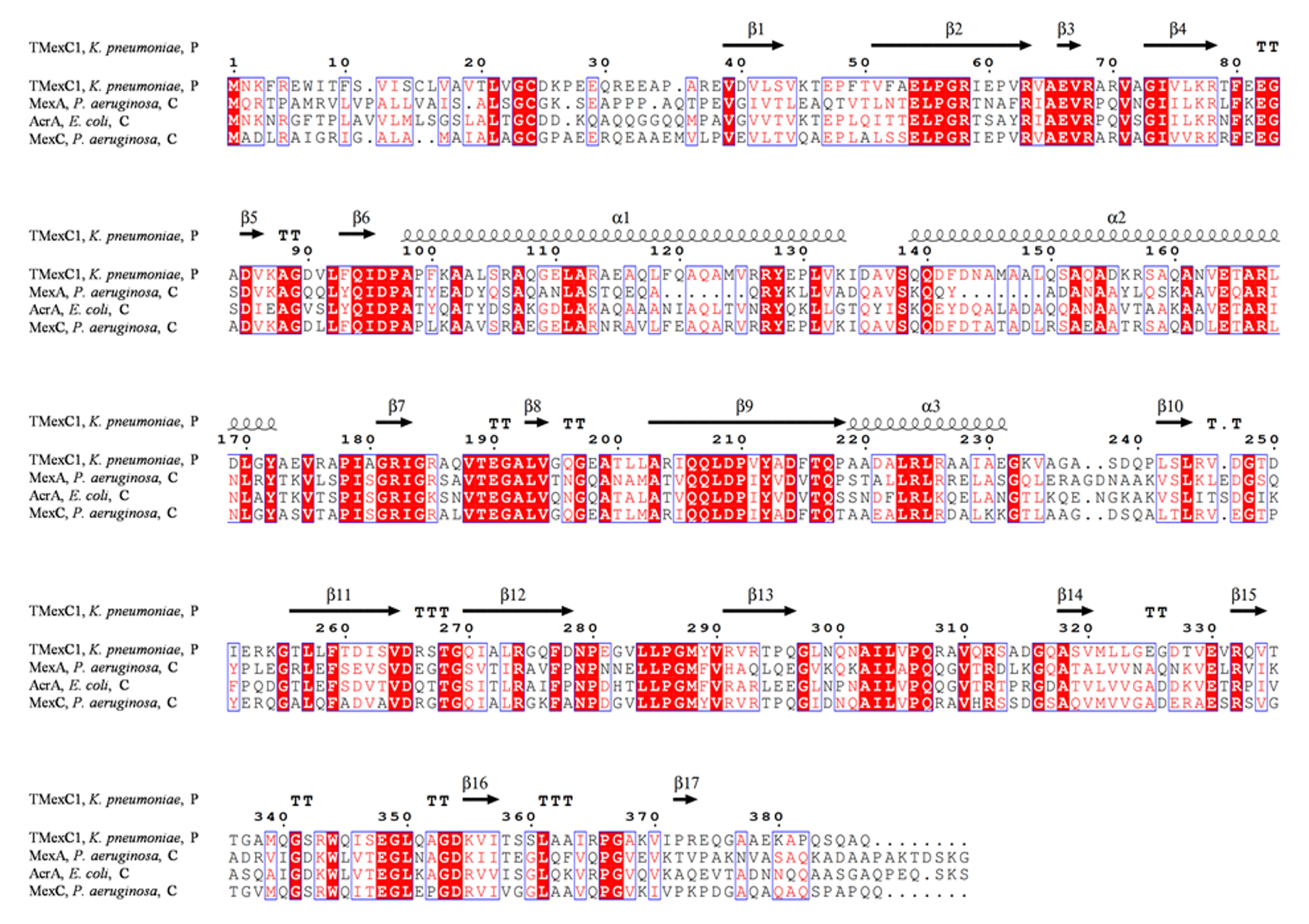

Supplement: FIG S2 [file mBio.02930-19-sf002.tif]

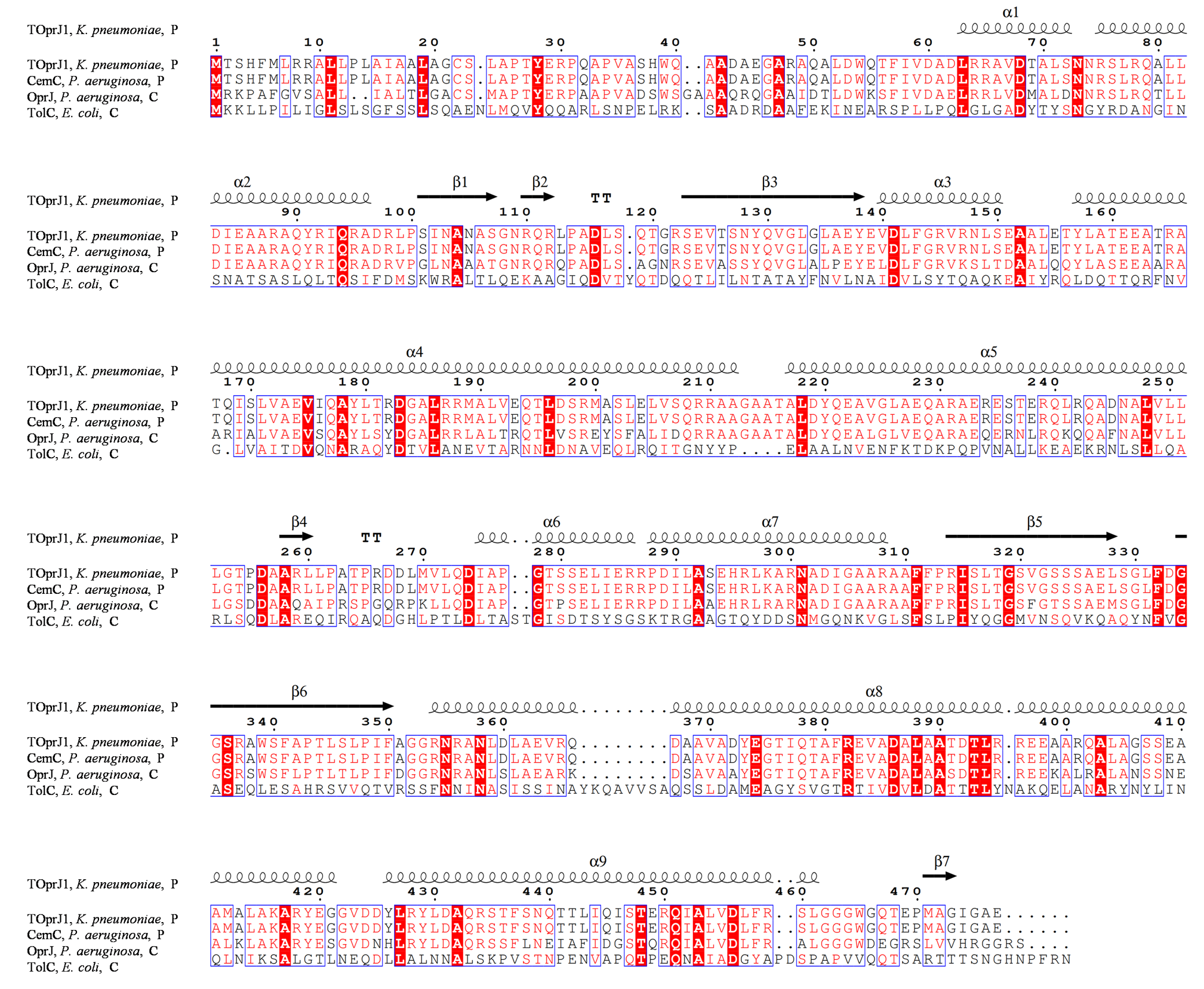

Supplement: FIG S4 [file mBio.02930-19-sf004.tif]
