# Supplementary material for: Emergence of a Plasmid-Encoded Resistance-Nodulation-Division Efflux Pump Conferring Resistance to Multiple Drugs, Including Tigecycline, in Klebsiella pneumoniae
Source: mBio. 2020 Mar 3;11(2):e02930-19. doi: 10.1128/mBio.02930-19 (PMC7064769; doi:10.1128/mBio.02930-19)
Supplement: TABLE S1 [file mBio.02930-19-st001.docx]

**TABLE S1** MICs against parental strain, transformants, and transconjugant (mg/liter).

| Strains | Ampcillin | Cefoxitin | Nalidixic acid | Amikacin | Chloramphenicol | Florfenicol | SXT ^a^ | Colistin^b^ | Olaquindox |
| --- | --- | --- | --- | --- | --- | --- | --- | --- | --- |
| *K. pneumoniae*AH8I, AH6I, AH25I, AH28I, AH33I | >128 | >128 | >128 | >128 | >128 | >128 | 32 | 64 | >128 |
| *E. coli* J53 | 4 | 2 | 4 | 0.5 | 4 | 4 | 0.25 | 0.125 | 8 |
| *E. coli* J53 + pHNAH8I-1 | 4 | 2 | 8 | 0.5 | 8 | 4 | 0.25 | 1 | 32 |
| *E. coli* DH5α | 4 | 2 | 2 | 0.5 | 4 | 2 | 0.25 | 0.125 | 2 |
| *E. coli* DH5α + pHNAH8I-1 | 8 | 8 | 32 | 2 | 16 | 8 | 0.25 | 1 | 128 |
| *E. coli* DH5α + pHSG575 | 8 | 2 | 2 | 0.5 | >128 | 2 | 0.25 | 0.125 | 1 |
| *E. coli* DH5α + pHSG575-tmexCD1 | 8 | 4 | 8 | 0.5 | >128 | 32 | 0.5 | 0.125 | 8 |
| *E. coli* DH5α + pHSG575-tnfxB1-tmexCD1 | 8 | 4 | 8 | 0.5 | >128 | 32 | 0.5 | 0.125 | 8 |
| *E. coli* DH5α + pHSG575-tmexCD1-toprJ1 | 8 | 8 | 16 | 1 | >128 | 32 | 0.5 | 0.125 | 16 |
| *E. coli* DH5α + pHSG575-tnfxB1-tmexCD1-toprJ1 | 8 | 8 | 16 | 1 | >128 | 32 | 0.5 | 0.125 | 16 |
| *K. pneumoniae* YX94 | 16 | 1 | 4 | 2 | 4 | 2 | 1 | 0.25 | 16 |
| *K. pneumoniae* YX94 + pHNAH8I-1 | 16 | 4 | 16 | 2 | 8 | 4 | 2 | >64 | 128 |
| *K. pneumoniae* YX94 + pHSG575 | 16 | 1 | 8 | 1 | >128 | 2 | 1 | 0.25 | 16 |
| *K. pneumoniae* YX94 + pHSG575- tnfxB1-tmexCD1-toprJ1 | 16 | 4 | 32 | 2 | >128 | 8 | 8 | 0.25 | 128 |
| *K. pneumoniae* AH58I | >128 | >128 | >128 | 2 | >128 | >128 | 32 | 0.25 | >128 |
| *K. pneumoniae* AH58I + pHNAH8I-1 | >128 | >128 | >128 | 2 | >128 | >128 | 32 | >64 | >128 |
| *S. typhimurium* HN227+ pHSG575 | 4 | 4 | 4 | 1 | >128 | 2 | 0.25 | 0.25 | 8 |
| *S. typhimurium* HN227+ pHSG575-tnfxB1-tmexCD1-toprJ1 | 16 | 16 | 32 | 2 | >128 | 8 | 2 | 0.25 | 128 |

^a^ SXT, trimethoprim-sulfamethoxazole.

^b^ MICs of colistin were determined by microdilution and of other antibiotics by agar dilution.
