# Supplementary material for: Emergence of a Plasmid-Encoded Resistance-Nodulation-Division Efflux Pump Conferring Resistance to Multiple Drugs, Including Tigecycline, in Klebsiella pneumoniae
Source: mBio. 2020 Mar 3;11(2):e02930-19. doi: 10.1128/mBio.02930-19 (PMC7064769; doi:10.1128/mBio.02930-19)
Supplement: TABLE S2 [file mBio.02930-19-st002.docx]

**TABLE S2** Isolates used in this study.

| Isolate | Species | Isolation time | Source | MLST | Characteristic(s)^a^ |
| --- | --- | --- | --- | --- | --- |
| **AH8I**,  AH25I, AH28I,  AH33I, AH6I | *K. pneumoniae* | 2017.5 | Chicken | ST1 | Resistant or reduced susceptibility to all antimicrobials, including TIG. |
| AH58I | *K. pneumoniae* | 2017.5 | Chicken | ST11 | Used as recipient; TIG^S^; *bla*_NDM-1_ on non-conjugative IncX3 plasmid lacking *tra* region. |
| YX94 | *K. pneumoniae* | 2016.10 | Pork | ST2906 | Used as recipient, susceptible to all antimicrobial agents tested. |
| HN227 | *S.* Typhimurium | 2014.7 | Porcine |  | Used as recipient, susceptible to all antimicrobial agents tested. |
| J53 | *E. coli* |  |  |  | Used as recipient, NaN_3_^R^ |

^a^ TIG, tigecycline.
