# Supplementary material for: Emergence of a Plasmid-Encoded Resistance-Nodulation-Division Efflux Pump Conferring Resistance to Multiple Drugs, Including Tigecycline, in Klebsiella pneumoniae
Source: mBio. 2020 Mar 3;11(2):e02930-19. doi: 10.1128/mBio.02930-19 (PMC7064769; doi:10.1128/mBio.02930-19)
Supplement: TABLE S3 [file mBio.02930-19-st003.docx]

**TABLE S3** Resistance genes and plasmids carried by *Klebsiella pneumoniae* AH8I

|  | Chromosome | Plasmids | | | | |
| --- | --- | --- | --- | --- | --- | --- |
|  |  | pHNAH8I-1 | pHNAH8I-2 | pHNAH8I-NDM | pHNAH8I-4 | pHNAH8I-5 |
| Size (bp) | 5,210,474 | 121,961 | 231,265 | 160,877 | 33,310 | 10,800 |
| Replicon type |  | FIA | FIB(K) | A/C (ST3) | X4 | Col440I |
| Resistance genes |  |  |  |  |  |  |
| Aminoglycosides |  | *strAB* | *aadA2*, *aph(3')-Ia*, *armA* | *aadA16*, *aac(3)-IId*, *aph(3')-Ia*, *strAB* |  |  |
| β-lactams | *bla*_SHV-1_ |  | *bla*_TEM-1b_, *bla*_DHA-1_ | *bla*_NDM-1_ |  | *bla*_TEM-1b_ |
| Colistin |  | *mcr-8.1* |  |  | *mcr-1* |  |
| Quinolones | *oqxAB* |  | *qnrB4* | *aac(6')-Ib-*cr |  |  |
| Fosfomycin | *fosA*^KP^ |  |  |  |  |  |
| MLS | *mdf(A)* |  | *mph*(A), *mph*(E), *msr*(E) | *mph*(A) |  |  |
| Sulphonamides |  |  | *sul1* | *sul1, sul2* |  |  |
| Trimethoprim |  |  | *dfrA12* | *dfrA27* |  |  |
| Phenicol |  |  |  | *catB3, floR* |  |  |
| Rifampicin |  |  |  | *arr3* |  |  |
| Tetracyclines |  | *tmexCD1-toprJ1* |  | *tet*(A) |  |  |

^a^, not determined.
