# Supplementary material for: Emergence of a Plasmid-Encoded Resistance-Nodulation-Division Efflux Pump Conferring Resistance to Multiple Drugs, Including Tigecycline, in Klebsiella pneumoniae
Source: mBio. 2020 Mar 3;11(2):e02930-19. doi: 10.1128/mBio.02930-19 (PMC7064769; doi:10.1128/mBio.02930-19)
Supplement: TABLE S4 [file mBio.02930-19-st004.docx]

**TABLE S4** Sites of infection by *K. pneumoniae*

| Origin | Number of isolates |
| --- | --- |
| Sputum^a^ | 1,018 |
| Urine | 445 |
| Blood | 267 |
| Gall Bladder^a^ | 249 |
| Peritoneal Fluid | 193 |
| Abscess | 118 |
| Bronchoalveolar lavage | 96 |
| Liver | 92 |
| Pancreas | 25 |
| Appendix | 17 |
| Thoracentesis | 13 |
| Stomach | 6 |
| Secretion | 5 |
| Bronchial brushing | 4 |
| Endotracheal aspirate | 4 |
| Pleural fluid | 3 |
| Colon | 2 |
| Small Intestine | 2 |
| Catheter | 1 |
| Rectum | 1 |
| Lung | 1 |
| Kidney | 1 |
| Others | 12 |
| **Total** | **2575** |

^a^one isolate from sputum (0.1%) and one isolate from gall bladder (0.4%) were positive for *tmexCD1-toprJ1*, giving a total of 2/2575 (0.08%).
