# Supplementary material for: Emergence of a Plasmid-Encoded Resistance-Nodulation-Division Efflux Pump Conferring Resistance to Multiple Drugs, Including Tigecycline, in Klebsiella pneumoniae
Source: mBio. 2020 Mar 3;11(2):e02930-19. doi: 10.1128/mBio.02930-19 (PMC7064769; doi:10.1128/mBio.02930-19)
Supplement: TABLE S5 [file mBio.02930-19-st005.docx]

**TABLE S5** Prevalence of food animal and retail meat samples carrying *tmexCD1-toprJ1*-positive *Enterobacteriaceae* isolates

| Source | Location | | Year | Number of samples | Number of positive samples (%) |
| --- | --- | --- | --- | --- | --- |
| Farm chicken | **Anhui** | Farm I | 2018 | 71 | 29 (40.85) |
|  |  | Farm II |  | 51 | 17 (33.33) |
|  |  | Total |  | 122 | **46 (37.70)** |
|  | **Shandong** | Farm I | 2018 | 60 | 38 (63.33) |
|  |  | Farm II |  | 60 | 50 (83.33) |
|  |  | Farm III |  | 60 | 15 (25.00) |
|  |  | Farm IV |  | 60 | 60 (100.00) |
|  |  | Farm V |  | 60 | 15 (25.00) |
|  |  | Farm VI |  | 60 | 19 (31.67) |
|  |  | Farm VII |  | 60 | 38 (63.33) |
|  |  | Total |  | 420 | **235 (55.95)** |
|  | **Henan** | Farm I | 2019 | 77 | 0 |
|  |  | Farm II |  | 170 | 76 (44.71) |
|  |  | Farm III |  | 75 | 2 (2.67) |
|  |  | Total |  | 322 | **78 (24.22)** |
|  | **Sichuan** | Farm I | 2019 | 75 | 7 (9.33) |
|  |  | Farm II |  | 91 | 34 (37.36) |
|  |  | Total |  | 166 | **41 (24.70)** |
|  | Guangdong | Farm I | 2019 | 89 | 11 (12.36) |
|  |  | Farm II |  | 98 | 2 (2.04) |
|  |  | Farm III |  | 74 | 0 |
|  |  | Total |  | 261 | 13 (4.98) |
|  | Hubei | Farm I | 2019 | 78 | 0 |
|  |  | Farm II |  | 97 | 0 |
|  |  | Farm III |  | 97 | 0 |
|  |  | Farm IV |  | 67 | 0 |
|  |  | Total |  | 339 | 0 |
|  | Liaoning | Farm I | 2019 | 50 | 0 |
|  |  | Farm II |  | 47 | 0 |
|  |  | Total |  | 97 | 0 |
|  | Chongqing | Farm I | 2019 | 80 | 0 |
|  | Jiangxi | Farm I | 2019 | 98 | 0 |
|  | Jiangsu | Farm I | 2019 | 421 | 0 |
|  | **Total** |  |  | **2326** | **413 (17.76)** |
| Farm pig | Guangdong | Farm I | 2019 | 63 | 0 |
|  |  | Farm II |  | 79 | 0 |
|  |  | Total |  | 142 | 0 |
| Chickens at slaughter | Guangdong |  | 2018 | 110 | 0 |
|  |  |  | 2019 | 286 | 4 (1.40) |
|  |  | Total |  | 396 | 4 (1.01) |
|  | Sichuan |  | 2019 | 181 | 6 (3.32) |
|  | Shandong |  | 2019 | 13 | 0 |
|  | Anhui |  | 2018 | 21 | 0 |
|  | Hunan |  | 2019 | 32 | 0 |
|  | **Total** |  |  | **643** | **10 (1.56)** |
| Chicken meat | **Shandong** |  | 2018 | 62 | 0 |
|  |  |  | 2019 | 50 | 1 (2.00) |
|  |  | Total |  | 112 | 1 (0.89) |
|  | Hunan |  | 2019 | 15 | 1 (6.67) |
|  | Anhui |  | 2018 | 72 | 0 |
|  | Sichuan |  | 2019 | 19 | 0 |
|  | **Guangdong** |  | 2018 | 20 | 0 |
|  |  |  | 2019 | 109 | 10 (9.17) |
|  |  | Total |  | 129 | 10 (7.75) |
|  | **Total** |  |  | **347** | **12 (3.46)** |
| Pork | Shandong |  | 2019 | 31 | 0 |
|  | Sichuan |  | 2019 | 38 | 0 |
|  | **Guangdong** |  | 2018 | 32 | 0 |
|  |  |  | 2019 | 171 | 11 (6.43) |
|  |  | Total |  | 203 | **11 (5.42)** |
|  | **Hunan** |  | 2019 | 80 | **1 (1.25)** |
|  | **Total** |  |  | **352** | **12 (3.41)** |
| **Meat** |  |  |  | **699** | **24 (3.43)** |
