# Supplementary material for: Emergence of a Plasmid-Encoded Resistance-Nodulation-Division Efflux Pump Conferring Resistance to Multiple Drugs, Including Tigecycline, in Klebsiella pneumoniae
Source: mBio. 2020 Mar 3;11(2):e02930-19. doi: 10.1128/mBio.02930-19 (PMC7064769; doi:10.1128/mBio.02930-19)
Supplement: TABLE S6 [file mBio.02930-19-st006.docx]

**TABLE S6** Primers used in this study.

| **Primer** | **Sequence （5'-3'）** | **Size (bp)** | **Purpose** |
| --- | --- | --- | --- |
| tmexC-F | TTCCGTGATCTCCTGTTTG | 880 | Detection of *tmexCD1-toprJ1* gene cluster |
| tmexC-R | GATGGCGTTCTGGTTGAG |  |  |
| tmexD-F | CAGCCAGGACTACAACTTC | 1314 |  |
| tmexD-R | TAGAGGAACTTCGGATTGC |  |  |
| repA-F | CAACAGTGGTGGCATCCT | 463 | Detect pHNAH8I-1 replicon to determine plasmid stability |
| repA-R | ATTGCTGCGAGAGTAGAGT |  |  |
| tnfxB1-tmexCD1-toprJ1-F | CAGAATGGTCTCGATGTCATC | 7430 | To constructpHSG575- tnfxB1-tmexCD1-toprJ1 |
| tnfxB1-tmexCD1-toprJ1-R | CGCTACTCAATCTCTGATGTCGG |  |  |
| pHSG575-tnfxB1-tmexCD1-toprJ1-F | GATGACATCGAGACCATTCTGCGTAATCATGGTCATAGCTGTTTCC | 3487 |  |
| pHSG575-tnfxB1-tmexCD1-toprJ1-R | CCGACATCAGAGATTGAGTAGCGCTCGAATTCACTGGCCGTCGTTTTAC |  |  |
| tnfxB1-deletion-F | GACTGGTTCCAATTGACAAGCGGCAATTCTCATCGGCAGCAGTTAAATCCA | 5133 | Deletion of tnfxB1 gene from pHSG575+tnfxB1-tmexCD1-toprJ1 to generate pHSG575 + tmexCD1-toprJ1 |
| toprJ1-connection-R | GTTTGTTCGACCAGGGCCATGCGGCGTAGCGCACCATCGCGGGTCAGGTAGG |  |  |
| toprJ1-connection-F | CCTACCTGACCCGCGATGGTGCGCTACGCCGCATGGCCCTGGTCGAACAAAC | 5349 |  |
| tnfxB1-deletion-R | TTGCCGCTTGTCAATTGGAACCAGTCATTCTTCCTGAACGGGGCTTGCAAC |  |  |
